# Recent Developments in the Adsorption of Heavy Metal Ions from Aqueous Solutions Using Various Nanomaterials

**DOI:** 10.3390/ma17215141

**Published:** 2024-10-22

**Authors:** Mahmoud M. Youssif, Heba G. El-Attar, Volker Hessel, Marek Wojnicki

**Affiliations:** 1 Faculty of Non-Ferrous Metals, AGH University of Krakow, al. A. Mickewicza 30, 30-059 Krakow, Poland; 2 Department of Chemistry, Faculty of Science, Tanta University, Tanta 31527, Egypt; heba.elattar@science.tanta.edu.eg; 3 School of Chemical Engineering, University of Adelaide, Adelaide 5005, Australia; volker.hessel@adelaide.edu.au; 4 School of Engineering, University of Warwick, Coventry CV4 7AL, UK

**Keywords:** heavy metals, wastewater, adsorption, metal oxide nanosorbents, functionalization, adsorption capacity

## Abstract

Water pollution is caused by heavy metals, minerals, and dyes. It has become a global environmental problem. There are numerous methods for removing different types of pollutants from wastewater. Adsorption is viewed as the most promising and financially viable option. Nanostructured materials are used as effective materials for adsorption techniques to extract metal ions from wastewater. Many types of nanomaterials, such as zero-valent metals, metal oxides, carbon nanomaterials, and magnetic nanocomposites, are used as adsorbents. Magnetic nanocomposites as adsorbents have magnetic properties and abundant active functional groups, and unique nanomaterials endow them with better properties than nonmagnetic materials (classic adsorbents). Nonmagnetic materials (classic adsorbents) typically have limitations such as limited adsorption capacity, adsorbent recovery, poor selective adsorption, and secondary treatment. Magnetic nanocomposites are easy to recover, have strong selectivity and high adsorption capacity, are safe and economical, and have always been a hotspot for research. A large amount of data has been collected in this review, which is based on an extensive study of the synthesis, characterization, and adsorption capacity for the elimination of ions from wastewater and their separation from water. The effects of several experimental parameters on metal ion removal, including contact duration, temperature, adsorbent dose, pH, starting ion concentration, and ionic strength, have also been investigated. In addition, a variety of illustrations are used to describe the various adsorption kinetics and adsorption isotherm models, providing insight into the adsorption process.

## 1. Introduction

**Figure 1:**
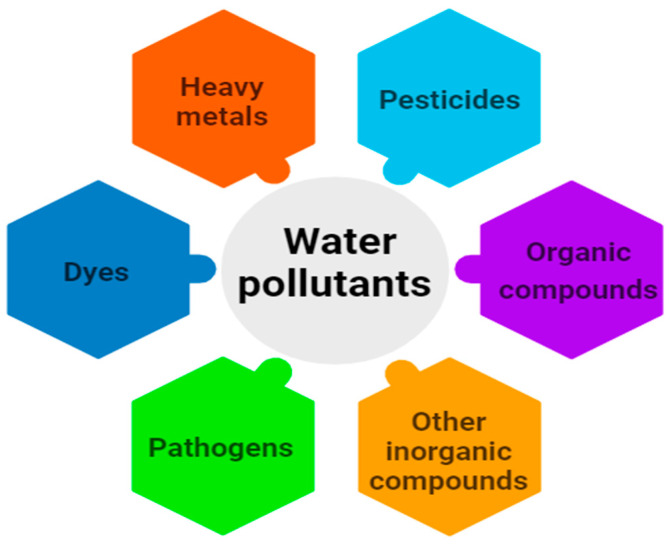
Different types of pollutants in wastewater.

The primary constituent of life on Earth is water. The amount and quality of water sources have been steadily declining due to urbanization, industry, and the expanding global population. Over 700 million people globally lack access to clean water due to various hazardous impurities caused by chemicals; industrial effluents, including inorganic and organic contaminants; and nuclear waste [1,2,3]. Figure 1 illustrates the various methods by which these compounds are released into the environment, including pesticides, fertilizers, metal complex dyes, fixing agents (which are used to increase the uptake of dye onto fibers), colorants, bleaching agents, heavy metals, and others.

In this review, we focus on water pollution by heavy metals. The atomic weights of heavy metals range from 63.5 to 200.6, and their specific gravities are greater than 5.0 [4]. There are two main types of sources of heavy metals: indirect and direct. The amount of heavy metals released into the environment is increasing due to the expansion of human activities and businesses, particularly in developing countries. This surge is attributed mainly to wastewater from various industries, including animal waste management, chemical manufacturing (for both industrial and agricultural purposes), mining operations, plating, and the production of paper and batteries [5,6]. Several heavy metals can change the physical, chemical, and biological properties of water and become cumulative, persistent, and nondegradable in the environment. They also pose a serious problem to human health and are known to cause cancer [7,8,9]. While some heavy metals are essential for human metabolism and important to life, such as those that keep vital enzyme sites working, others can be extremely harmful to living organisms [10,11]. International authorities, including the WHO, FAO, and EPA, consistently monitor the effects of these ions on the health of humans [12,13]. The most common heavy metals found in various industries are arsenic (As), cadmium (Cd), chromium (Cr), copper (Cu), lead (Pb), mercury (Hg), zinc (Zn), and Uranium U(VI). Table 1 lists the sources, effects, and allowable limits of certain heavy metals on human health [14,15,16,17,18,19,20,21,22,23,24].

Eliminating dangerous heavy metals with high efficiency from industrial and wastewater is the best solution option [25,26,27]. Various methods have been utilized for purifying water to eliminate these pollutants from wastewater, including methods such as solvent extraction, membrane filtering, chemical precipitation, and adsorption [28]. Ultimately, the most efficient method is adsorption, which has the greatest potential for eliminating metal ions from water. Adsorption is the process by which an atomic or molecular film is formed on the surface of an adsorbent (liquid or solid) by a solute (gas or liquid) called an adsorbate. This method works well in many different systems, including chemical, physical, biological, and industrial systems [29]. Owing to its low cost, reversibility, ease of use, and other noteworthy benefits, it has been demonstrated for many years to be the most effective wastewater treatment method [30,31]. Furthermore, the use of recyclable adsorbents makes the adsorption process extremely economical [32]. Owing to these characteristics, researchers are paying careful attention to the adsorption process as a way to remediate heavy metal ion-containing industrial effluent [33]. Limited adsorption capabilities, nonfunctional tunability, nonrecyclability, and reusability are some of the disadvantages of these conventional adsorbents. Several nanoscale sorbents are being created and used to purify water to overcome these limitations. Since nanoparticles have a very large specific surface area and several active groups that can bind heavy metal ions, there is currently much interest in employing them as adsorbents in wastewater purification [34]. Additionally, the cost-effectiveness and attractiveness of nanostructured adsorbents comes from their ability to be recovered and reused repeatedly. However, their irregularity, difficulty in being removed from treated water, and regeneration have limited their commercial application [35]. As a result, scientists are working to functionalize NPs to solve these problems [36]. The adsorption capacity is increased and separation is made easier with appropriate surface functionalization.

**Figure 2:**
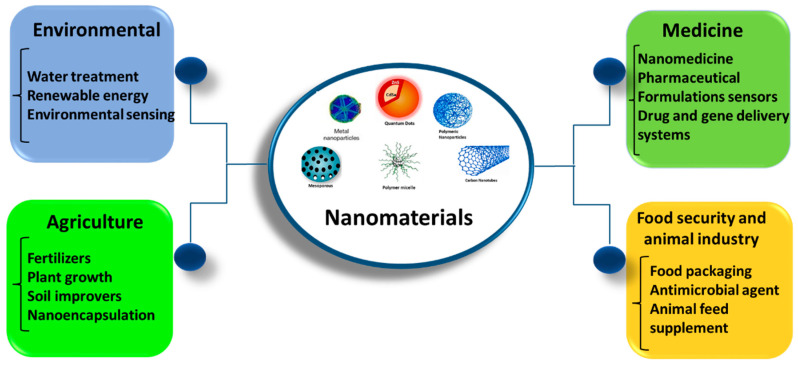
Applications of nanomaterials in environmental, agricultural, medical, food, and animal industries.

This review examined various nanoadsorbents that have been recently developed to extract chromium, cadmium, and copper ions from wastewater [37,38,39,40,41,42]. One of the highest-quality substitutes for heavy metal adsorption is nanomaterials. The application of nanomaterials is highly desirable in the fields of environmental problems [43], energy and storage devices [44], metal protection from corrosion [45], optoelectronics [46], analytical sciences [47], and biological sectors [48]. Figure 2 illustrates how nanomaterials are used in several fields. Recently, nanomaterials have been increasingly used to treat water because of their simplicity of synthesis and functionalization, high active surface area, chemical diversity, thermal and mechanical stability, and physical tunability. Compared with traditional materials, they are categorized as photocatalytic materials, adsorbents, membranes, etc. [49].

A nanomaterial is said to be an effective adsorbent if it is nontoxic, has high absorptivity, uses less energy, is recyclable, and can be reused [50]. There are various categories of nanomaterials, such as nanoparticles, nanotubes, nanowires, nanolayers/sheets, and nanospheres. These include metal oxide nanoparticles, carbon-based nanomaterials, and nanocomposites. Previously, a variety of NP types, such as metallic, metal oxide, bimetallic, magnetic, ferrite, natural and synthetic polymers, and porous materials, were investigated for their potential to purify wastewater [51]. These nanomaterials eliminate heavy metal ions mainly by adsorbing them onto their surfaces. The several varieties of NPs that have been confirmed in recent scientific studies for previous reasons are summarized in detail below.

The different types of pollutants, their effects on human health, the factors influencing the adsorption phenomenon, and the use of biological materials and nanomaterials such as metals, metal oxides, magnetic nanocomposites, and carbon nanomaterials as adsorbents for the efficient removal of heavy metals through adsorption techniques are covered in this review. Different nanomaterials have been used to adsorb heavy metals, and several adsorption isotherms have been discussed. In addition, current studies have concentrated on how to modify nanomaterials to increase their separation from the suspension medium and dispersion stability, hence increasing their efficacy in the elimination of toxic metal ions. Nanoadsorbents are functionalized with different types of compounds, including inorganic, organic, polymeric, and biomolecular materials, etc. Based on functionalized nanomaterials, research has proven to be helpful and dependable. As such, it might open the door for systems related to the surroundings.

## 2. Nanomaterials as Adsorbents Applied for the Removal of Metal Ions from Water

**Figure 3:**
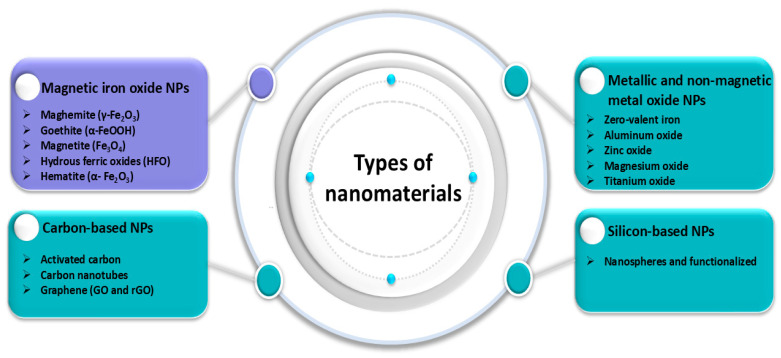
Different types of nanomaterials for heavy metal removal in aqueous media.

Nanomaterials (NMs) have been extensively studied for wastewater treatment over the past two decades because of their remarkable properties, including high specific surface area, porosity, surface functionalities, and ion binding capabilities. In fact, the use of these materials as adsorbents has significant potential for the removal of metallic ions even at tiny levels. Nanomaterials are divided into two categories: (i) nonmagnetic nanomaterials, such as zero-valent iron (ZVI); nonmagnetic metal oxide nanoparticles, such as magnesium oxide, aluminum oxide, zinc oxide, and titanium oxide; silica and carbon-based nanomaterials; and (ii) magnetic iron oxide-based nanomaterials and nanocomposites, as shown in Figure 3.

### 2.1. Nonmagnetic Nanomaterial-Based Nanoadsorbent

#### 2.1.1. Metallic Nanomaterials

The most significant metallic nanoparticle (NP) is nano zero-valent iron (nZVI) because of its superiorsorption capacity, enhanced surface area, nontoxicity, and excellent stability. Zero-valent iron has been shown in numerous studies to be the most important material for eliminating metal ions from the environment. Azzam et al. [52] synthesized iron-based nanoparticles (NPs) from FeCl_3_ via a reduction method. The maximum removal capability of the generated NPs (133, 181.8, 153.8, and 1250 mg g^−1^) was shown to be very successful in eliminating Ni^2+^, Cu^2+^, Cd^2+^, and Pb^2+^ ions, respectively. According to a different study by Li et al. [53], nZVI can remove different types of heavy metal ions quickly and simultaneously, including As^5+^, Cu^2+^, Zn^2+^, and Ni^2+^ ions. In a related study, Boparai et al. demonstrated that synthesized zero-valent iron NPs could remove Cd^2+^ ions [54] with maximum adsorption capacity. nZVI was trapped in a biodegradable and nontoxic stabilizer, such as the chitosan carboxymethyl β-cyclodextrin complex, by Sikder et al. [55]. The synthesized material was used to remove Cr^6+^ and Cu^2+^ ions by physical sorption followed by the reduction of C^6+^ to Cr^3+^ and of Cu^2+^ to Cu^o^ while oxidizing Fe^o^ to Fe^3+^. As documented in existing research, bimetallic nanoparticles have been used in heavy metal ion elimination. In this regard, bimetallic Fe/Ni NPs embedded in kaolinite (K-Fe/Ni) were synthesized by Cai et al. [56]. Cu^2+^ ions could be removed simultaneously with these Fe/Ni NPs, with removal efficiencies of 99.7%. Furthermore, a new porous (styrene–divinylbenzene)/CuNi bimetallic nanocomposite microsphere was synthesized according to Hridya et al. [57]. The efficacy of the generated nanocomposite for the removal of Pb^2+^, Cd^2+^, Mn^2+^, and Zn^2+^ ions from synthetic water was tested via batch adsorption research. The best pH range for lead adsorption was found to be 5–7, but the ideal pH range for zinc, cadmium, and manganese adsorption was found to be 7. In the past few years, bare metal nanoparticles have become less frequently used as adsorbents because removing bare nanoparticles from wastewater is difficult. Therefore, the trend has been toward the use of magnetic materials to increase the removal efficiency, ease of separation, and reuse of metals more than once.

#### 2.1.2. Magnesium Oxide-Based Nanoadsorbent

The best option for treating wastewater via an adsorption process has been shown in recent years to be nanostructured metal oxides [58]. The role of metal oxide nanoparticles (NPs), such as iron, titanium, zinc, and aluminum oxides, in wastewater decontamination has been demonstrated in a plethora of scientific investigations conducted by different research teams, as Figure 3 illustrates. They are divided into nonmagnetic and magnetic metal oxide nanoparticles (NPs) according to their inherent magnetic properties. Cu, Mn, Zn, Al, Ce, and other oxides are examples of nonmagnetic NPs that have been used to eliminate heavy metal ions [59,60,61,62]. Different alkaline metal oxide nanoparticles have also been utilized in addition to transition metal oxide NPs for the purpose of removing metal ions. They are thought to be less harmful and environmentally favorable compared with metal oxide nanoparticles.

Significant members of this category, MgO NPs, have been studied by numerous researchers and shown to be efficient in removing heavy metal ions. Using flower-like MgO NPs with a wide surface area as an example, Cao et al. [63] demonstrated exceptional removal effectiveness for Cd^2+^ and Pb^2+^ ions, with maximal capacities of 1500 mg g^−1^ and 1980 mg g^−1^, respectively. The remarkable adsorption capabilities were attributed to a novel adsorption mechanism that involved cation exchange between Mg^2+^ ions from MgO NPs and Pb^2+^ or Cd^2+^ ions. In accordance with Qin et al., MgO NP-modified TOBs were used to purify wastewater containing Cd^2+^ and Cu^2+^ ions. When combined with TOB, the generated MgO NPs effectively removed Cd^2+^ and Pb^2+^ ions [64]. In a different study, Wei et al. [65] reported that porous Mg-infused chitosan beads underwent pyrolysis to create MgO-embedded granular hierarchical porous biochar (HP-MgO@BC). HP-MgO@BC showed remarkably fast adsorption of Pb^2+^ and Cu^2+^ ions. The sorption capacities of Pb^2+^ and Cu^2+^ were 1044.8 mg g^−1^ and 811.2 mg g^−1^, respectively. High adsorption efficiency was demonstrated by HP-MgO@BC in the presence of interfering cations, enabling the removal of Pb^2+^ and Cu^2+^ over a wide pH range (2–7). Its reusability was equally exceptional.

#### 2.1.3. Aluminum Oxide-Based Nanoadsorbent

**Figure 4:**
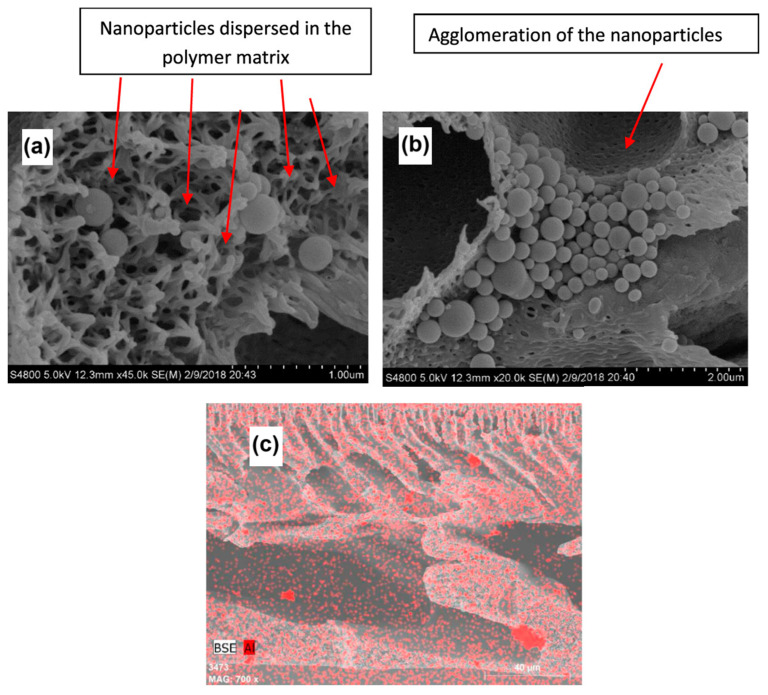
Higher magnification SEM cross-sectional image of (**a**) M4 membrane showing incorporation of the nanoparticles in the polymer matrix; (**b**) M5 showing the agglomeration of nanoparticles; and (**c**) energy-dispersive X-ray map scanning spectra for the cross-section of the M4 sample. Figure reproduced with permission of [68]. Copyright 2018, American Chemical Society.

Alumina (Al_2_O_3_), another essential metal oxide, may be found in natural soils and occurs in a variety of structural phases, including α, β, γ, θ, and χ. α-Al_2_O_3_ is utilized as a naturally occurring adsorbent with increased stability [66]. Strong interatomic bonding makes alumina an attractive sorbent because of its interesting features, which include compressive strength, good electrical insulation, thermal conductivity, and wear and corrosion resistance [67]. Researchers have utilized Al_2_O_3_ NPs to promote the adsorption of particular nanomembranes. Gohari et al. applied γ-Al_2_O_3_ nanoparticles to increase the removal efficacy of copper ions for polyethersulfone (PES) membranes. In this case, the APTES silane coupling agent was first used to alter the alumina nanoparticles before they were injected into the PES composite membranes. Through the use of phase inversion, three distinct concentrations of modified nanoparticles were incorporated into the PES membranes. Several measurements were used to characterize the prepared membranes. The ability of the membranes to remove Cu^2+^ ions and their adsorption capacity were also examined. The membrane surface area, glass transition temperature, overall porosity, hydrophilicity, and thermal stability are all enhanced with the inclusion of nanoparticles. TGA verified that the nanoparticles were appropriately absorbed throughout the membrane fabrication process. Additionally, there was a notable increase in membrane water penetration. As shown in Figure 4a, for the M4 membrane sample, the morphology of the membranes and the presence and dispersion of nanoparticles in the membrane structure were also examined via SEM. The suitable dispersion of alumina nanoparticles in the membrane matrix was confirmed, as shown in Figure 4c. The even distribution of nanoparticles is beneficial because it enhances the contact area between the water passing through the membrane and the particle surfaces, thereby increasing the surface adsorption of copper ions. Interestingly, the M5 samples have smaller surface areas than the M4 and M3 samples. This is explained by the nanoparticles clumping together, as Figure 4b illustrates. Agglomeration may also cause some structural pores to become blocked, which would reduce the available surface area of the membrane. The combination of higher porosity and lower hydrophobicity of the membrane surface led to a significantly greater water flux. Furthermore, in the case of pure polymer membranes, copper ion removal increased from 11% to 87% for nanocomposite membranes containing a 4% weight percentage of modified alumina nanoparticles [68]. In another investigation, Hojamberdiev et al. [69] incorporated benzimidazole and dithizone into the pores of Al_2_O_3_ nanoparticles, filling 25% of the total pore volume. These new solid-phase sorbents were examined for their ability to adsorb metal ions (Ni^2+^, Cd^2+^, and Zn^2+^) from contaminated water. The prepared sorbents exhibited the highest sorption capacities for Ni^2+^ (0.37 mmol g^−1^), Zn^2+^ (0.54 mmol g^−1^), and Cd^2+^ (1.01 mmol g^−1^). The physisorption and chemical interaction between the metal ions and the nanocomposites resulted in the sorption of these heavy metal ions.

The adsorption performance of the Al_2_O_3_ nanoparticles was improved by the functionalization and modification of their surface. The detection limit and relative standard deviation of Al_2_O_3_ modified with dimethylglyoxime (DMG) and sodium dodecyl sulfate (SDS) are 1.9% and 0.06 µg L^−1^, respectively, under optimal circumstances. Furthermore, 365 mg g^−1^ had the highest adsorption capacity of the synthesized Al_2_O_3_/SDS–DMG for adsorbing palladium ions. The research findings demonstrate that the suggested approach has the potential to be used for determining Pd^2+^ ions in trace quantities in real samples, such as tap water [70]. In another study, the adsorption of α-Al_2_O_3_ modified with sodium dodecyl sulfate (SDS) to NH_4_^+^ from aqueous solution was found to be enhanced, resulting in very high removal efficiencies of 99.5 and 96.5% under optimum conditions [71]. Furthermore, Nguyen et al. [72] demonstrated the effective removal of Cd^2+^ ions using both sodium tetradecyl sulfate and sodium dodecyl sulfate-modified γ-Al_2_O_3_ nanoparticles. The elimination effectiveness dramatically increased from 67% to 94.6% when the impact of the modified and unmodified γ-Al_2_O_3_ nanoparticles was examined.

#### 2.1.4. Zinc Oxide-Based Nanoadsorbent 

ZnO nanoparticles have been successfully used to eliminate metals from water and wastewater because of their mechanical properties, thermal durability, high surface area at room temperature, and superior elimination ability [73]. Nanoparticles are promising for use in drug delivery studies, solar cells, capacitors, gas sensors, and catalysis in addition to wastewater purification studies because of their high chemical stability, pyroelectric and piezoelectric properties, and other properties [74]. ZnO nanoparticles also have adjustable morphological features and easy, less expensive production. Consequently, a variety of techniques have been used to fabricate ZnO particles at the nanoscale. Zinc precursors such as ZnCl_2_, Zn(NO_3_)_2_·6H_2_O, Zn(SO_4_)_2_·7H_2_O, and Zn(C_2_H_3_O_2_)_2_·2H_2_O have all been used to date for the synthesis of ZnO nanoparticles. Most notably, hydrolysis and condensation are hindered when ZnO is synthesized in media with a pH of less than 7 (low OH^−^ concentration). This may therefore result in poorer crystallization and the formation of aggregates. Owing to the equivalent OH^−^ and H^+^ concentrations in a neutral medium (pH = 7), the solution combination may have contributed little to no intense contribution at the ZnO crystal interfaces [75]. Because of the relatively high concentration of OH^−^, which electrostatically attracts incoming positively charged Zn^2+^ ions and may cause small ZnO nanoparticles and crystallization, the ideal pH for the formation of ZnO nanoparticles is pH > 7 [76]. The size, shape, surface area, and phase composition of the synthesized ZnO nanoparticles are also influenced by the calcination temperature, incubation duration, and precursor and surfactant concentrations. By using *Centaurea cyanus* extract (biosynthesis) at room temperature, ZnO nanoparticles with an average particle size of 49 nm were synthesized. Adsorption period = 91.25 min, adsorbent dose = 1.63 g/L, and Pb^2+^ ion concentration = 77.5 mg/L were the ideal adsorption conditions for Pb^2+^ ions. At a pH of 5.5, the highest adsorption of 99.24% was observed experimentally. The equilibrium adsorption data isotherm suggested that the ZnO nanoparticle surface had a heterogeneous distribution of accessible and active adsorption sites. Subsequent adsorbate coverage produced a multilayer adsorption system [77]. In a related study, ZnO nanoparticles (NPs) were manufactured by Azizi et al. in an environmentally friendly manner, and their capacity to absorb Pb^2+^ ions from aqueous solution was evaluated. In batch investigations, the effects of process variables on the Pb^2+^ concentration, adsorbent mass, temperature, pH, and contact duration on the Pb^2+^ elimination efficiency were examined. The adsorption process was spontaneous and endothermic, with a maximum removal efficiency of 93% at pH 5. The highly effective nature of the synthesized NPs makes them interesting for application in water treatment cases where the elimination of heavy metals from aqueous solutions is crucial [78].

#### 2.1.5. Titanium Oxide-Based Nanoadsorbent

TiO_2_ nanoparticles are affordable, easily synthesized, photocatalytic, and chemically stable, making them promising options for eliminating harmful metals from synthetic aqueous solutions and wastewater. Unlike brookite, which is necessary for industrial applications, the phase compositions that are frequently utilized include rutile and anatase [79]. Remarkable Cd^2+^ and Pb^2+^ ion removal from aqueous solutions was achieved via mesoporous ZnO and TiO_2_@ZnO monoliths, which were synthesized via a nanocasting process [80]. The results indicated that the maximum adsorption efficacies of Pb^2+^ ions for the ZnO and TiO_2_@ZnO monoliths were 790 and 978 mg L^−1^, respectively. On the other hand, the maximum adsorption efficacies of Cd^2+^ ions for the ZnO and TiO_2_@ZnO monoliths were 643 and 786 mg L^−1^, respectively. Additionally, the mesoporous ZnO and TiO_2_@ZnO monoliths may be effectively reused a minimum of three times following NaOH treatment. The excellent adsorption capacity of the ZnO and TiO_2_@ZnO monoliths for Pb^2+^ and Cd^2+^ ions, together with their remarkable reusability, make them potentially appealing adsorbents. In another study, phosphate-modified titanium dioxide nanoparticles with a mesoporous structure were synthesized and utilized as efficient materials for eliminating Sr^2+^ ions from aqueous environments. Because of the significant number of surface acid sites, a mesoporous material based on titanium dioxide has notable adsorption activity. According to all of these results, phosphate-modified titania is promising for removing Sr^2+^ ions from aquatic environments [81]. According to Ezati et al. [82], TiO_2_ and γ-Al_2_O_3_ nanoparticles have been produced as adsorbents to eliminate copper ions from aqueous solutions. At pH 8, the highest monolayer adsorption capacity of the TiO_2_ and γ-Al_2_O_3_ nanosorbents (9288 and 3607 mg kg^−1^, respectively) and the highest initial concentration of Cu^2+^ ions (80 mg L^−1^) were observed. The adsorption efficiency for TiO_2_ and γ-Al_2_O_3_ nanoparticles increased as the pH increased and decreased as the ionic strength increased. When TiO_2_ nanoparticles at 0.01 M ionic strength were used, the greatest amount of Cu^2+^ ions adsorbed (4510 mg kg^−1^) was obtained in the presence of NaCl. Thermodynamic simulations demonstrated that the adsorption of Cu^2+^ ions onto the nanoparticles was spontaneous. As a result, TiO_2_ nanosorbents may be used as practical a(nd affordable nanomaterials to clean aqueous solutions contaminated with Cu^2+^ ions.

#### 2.1.6. Silica-Based Nanoadsorbent

**Figure 5:**
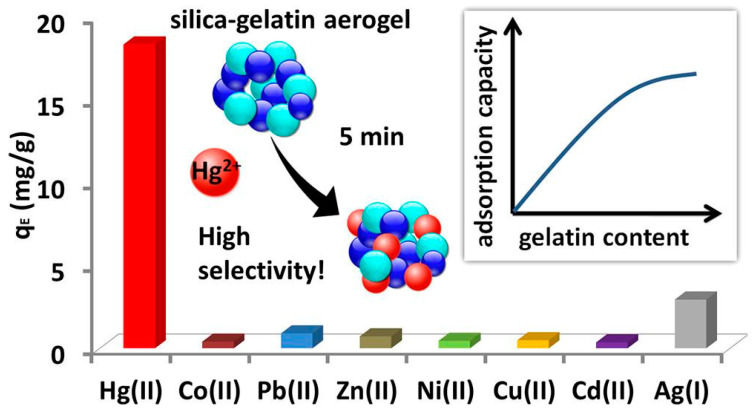
Mesoporous silica–gelatin hybrid aerogels exhibit enhanced selectivity in the adsorption of aqueous Hg^2+^ ions in comparison to other meta ions. Furthermore, the adsorption uptake of these hybrid aerogels rises as the gelatin content increases. Figure reproduced with permission of [86]. Copyright 2020, American Chemical Society.

A material based on nanostructured silica is being developed to adsorb metal ions because of its large surface area, varied surface characteristics, and typical pore diameter. Additionally, because nano silica is chemically inert and has no negative health consequences, it is a nontoxic, ecologically friendly adsorbent. Adsorption sensitivity and such major restrictions are made possible by chemical nano silica modifications, including amino–thiol combinations. Additionally, the adsorption capability and selectivity are enhanced when amino and thiol functional groups are added to the surface of nano silica. Using nitrilotriacetic acid-modified silica gel (NTA silica gel), Yulian Li et al. [83] eliminated Cd^2+^, Pb^2+^, and Cu^2+^ ions from wastewater. The NTA silica gel demonstrated fast removal of three metal ions, Pb^2+^, Cu^2+^, and Cd^2+^ ions, with comparatively high adsorption capacities (53.14, 63.5, and 76.22 mg g^−1^ for Cd^2+^, Cu^2+^, and Pb^2+^ ions, respectively) and removal efficiencies ranging from 96% to 99%. The NTA silica gel removal efficacy improved throughout a broad pH range (2–9) and remained stable when competing metal ions (Na^+^, Mg^2+^, Ca^2+^, and Al^3+^ ions) were present at various concentrations. In addition, the NTA silica gel was easily regenerated and reused for five cycles, resulting in a high adsorption capacity. This study indicates that NTA silica gel is a reusable adsorbent for the rapid, convenient, and efficient removal of Cu^2+^, Cd^2+^, and Pb^2+^ ions from contaminated aquatic environments. The use of silica nanospheres functionalized with amino groups was studied in a different study to improve the removal of Cu^2+^ ions [84]. The adsorption ability increased with increasing amino group content. Additionally, Najafi et al. [85] provided information on the comparative removal of Ni^2+^, Pb^2+^, and Cd^2+^ ions by utilizing different amino-functionalized materials. Greater adsorption ability was demonstrated for functionalized nano silica than for nonfunctionalized nano silica. Cd^2+^, Ni^2+^, and Pb^2+^ ions had maximum adsorption capacities of 40.73, 31.39, and 96.79 mg g^−1^, respectively, when amino-functionalized silica hollow spheres were used. In another study, mesoporous silica–gelatin hybrid aerogels with 4–24 weight percent gelatin content, according to Herman et al. [86], exhibited high selectivity for the adsorption of aqueous Hg^2+^ ions in the presence of Cu^2+^, Cd^2+^, Co^2+^, Pb^2+^, Ni^2+^, Ag^+^, and Zn^2+^ ions, as shown by batch adsorption experiments with multiple competing ions in Figure 5. The Hg^2+^ ion adsorption ability of the hybrid aerogels increased with increasing gelatin content, reaching a plateau at 24 wt% gelatin. The 24 wt% gelatin hybrid is anticipated to have an adsorption capacity of 209 mg g^–1^. An increased gelatin content leads to increased adsorption capability because gelatin supplies the active sites for Hg^2+^ binding. Nevertheless, a high gelatin content also encouraged the backbone to swell significantly, and the open porous structure partially collapsed, reducing the specific surface area. According to time-resolved investigations, 15 min of contact time with aqueous Hg^2+^ is sufficient to achieve adsorption equilibrium. By washing the equilibrated aerogels with EDTA as a complexing agent, the quantitative release of bound Hg^2+^ ions can be achieved. After five rounds of reuse, the regenerated aerogels still have approximately full adsorption capabilities. Studies based on coordination chemistry indicate that the soft Lewis base side chains of collagen preferentially complex with Hg^2+^ ions.

Furthermore, an additional scientific study [87] described the synthesis of a unique silica gel/graphene oxide nanocomposite (SG@GO-IIP) and how it can be used to recover palladium from a commercial industrial catalyst. Various approaches have been employed to characterize the nanocomposite material, and a potential adsorption mechanism between Pd^2+^ ions and SG@GO-IIP has been proposed. The findings indicated that adsorption equilibrium was attained in approximately 90 min, and the highest adsorption capacity reached 154.3 mg g^−1^. With its maximum adsorption capacity decreasing by only 8.9% after five adsorption/desorption cycles, the SG@GO-IIP material demonstrated good regeneration and reusability properties and excellent affinity towards Pd^2+^ ions even in the presence of K^+^, Na^+^, Ca^2+^, Mg^2+^, and Al^3+^ ions. The adsorption mechanism of Pd^2+^ ions onto SG@GO-IIP is considered to involve the formation of coordination bonds between the Pd^2+^ ions and iodine in the adsorbent. This research suggests a novel approach for recovering palladium from a spent industrial catalyst through solid-phase extraction, employing SG@GO-IIP as a sorbent. Sulfuric acid was selected as the leaching liquor in the procedure, and the technology could enable the utilization of graphene oxide-based nanocomposite materials for industrial applications.

#### 2.1.7. Carbon-Based Nanomaterials

Owing to the exceptional mechanical, thermal, and electrical properties of carbon, its nanoparticles are desirable choices for use in a range of applications, such as electronics and medicine delivery [88]. Furthermore, because of their many qualities, such as their high sorption capabilities, broad surface area, nontoxicity, and affinitive behavior, carbon materials are well suited for the removal of inorganic, organic, and pathogenic contaminants from wastewater bodies [89].

- Activated carbon

The effectiveness of activated carbon can be increased by increasing its surface area and pore size. Heavy metals were eliminated from wastewater in multiple tests by using activated carbon. The price of activated carbon (AC) has increased as a result of a shortage of commercial AC. The large surface area, high porosity, and adaptability of activated carbon make it a suitable candidate for wastewater treatment [90]. Although coal is used mainly to produce activated carbon, carbon adsorbents are often derived from carbonaceous sources such as biomass, lignite, and coal, among others. To remove cobalt and cadmium ions from wastewater, Tounsadi et al. [91] synthesized activated carbon from a novel biomass source (*Glebionis coronaria* L.). The carbonization temperature, activation temperature, activation period, and impregnation ratio are some of the factors that affect the generation of activated carbon in this research. The elimination of cobalt and cadmium ions was enhanced by the interaction between the impregnation ratio and the carbonization temperature. Consequently, the best sorption capacities were 45.75 mg g^−1^ cobalt and 57.87 mg g^−1^ cadmium [91]. Activated carbon can also be produced from a variety of agricultural byproducts, according to some researchers. Lead ions were extracted from an aqueous solution, for example, by using European black pine as a precursor in the manufacture of activated carbon. The adsorption capacity of activated carbon is 27.53 mg g^−1^ at an ideal concentration of 2.0 mg/L, according to the Langmuir model [92].

To improve the adsorption efficiency of activated carbon materials for heavy metals, some researchers have modified them by adding nanoparticles, adding functional and nitrogen groups, and adding anionic surfactants to their surface. A recent study by a scientist used an oxidation technique to modify the surface of AC, which increased the adsorption effectiveness in the elimination of heavy metals [93]. Using activated carbon filters oxidized by ammonium persulfate (APS) solutions, they studied the removal of Pb^2+^ ions from aqueous solutions. The maximum sorption capacity was 559 mg g^−1^ [93]. Similarly, to increase the adsorption capacity and affinity for metals, Chen et al. [94] extracted activated carbon (AC) from waste wood-based panels as the basic material, oxidized it with nitric acid (OAC), and grafted it with iminodiacetic acid (IDA-OAC). An increase in the number of modified adsorbent functional groups was demonstrated via instrument examination. Additionally, studies using batch and column methods were used to assess the adsorbent’s capacity for eliminating copper ions from aqueous solutions. At pH 5, IDA-OAC had the maximum adsorption capacity (84.51 mg g^−1^) for batch sorption compared with OAC (54.74 mg g^−1^) and AC (24.86 mg g^−1^). The excellent application of IDA-OAC for water treatment was demonstrated by its notable capacity and reusability. Furthermore, Sharmaand et al. [95] synthesized and studied a composite of datestone-activated carbon-based zirconium oxide (DSAC/ZrO_2_). It has been shown that the composite performs well as an adsorbent to remove cadmium ions from water systems. The greatest adsorption capacity was 166.7 mg g^−1^ at 25 °C and 200 mg g^−1^ at 40 °C. All of the adsorption investigations demonstrated the potential of the DSAC/ZrO_2_ composite as an adsorbent for the removal of hazardous cadmium heavy metal ions. The potential of the DSAC/ZrO_2_ composite as an adsorbent for organic pollutants and other heavy metal ions may be explored in future studies. The addition of metal/metal oxide nanoparticles enhances activated carbon, which increases its ability to adsorb the metal ions found in wastewater. These nanoparticles act exclusively against certain kinds of contaminants that are present in contaminated water.

ii Carbon nanotubes

The outstanding properties and applications of carbon nanotube (CNT) adsorbents make them a popular choice for heavy metal treatment procedures. Chemical vapor deposition (CVD) is a common method used to produce CNTs. Additionally, carbon nanotubes are divided into two types: multiwalled carbon nanotubes and single-walled carbon nanotubes with widths of 100–1000 nm and widths of 1–3 nm [96]. Numerous studies have shown the effectiveness of using CNTs to extract heavy metal ions from wastewater. For example, the following sequence of ion removal was achieved adsorptively using CNTs. According to Stafiej et al. [97], the amount of Cu^2+^ removal was greater than that of Pb^2+^, Co^2+^, Zn^2+^, and Mn^2+^ ions. In another study, MnO_2_-coated oxidized multiwalled carbon nanotubes (MnO_2_/MWCNTs) had an adsorption capacity of 41.6 mg g^−1^ and successfully removed Cd^2+^ ions from an aqueous solution [98]. However, owing to their strong accumulation and lack of functional groups, CNTs are used less frequently. Certain modifications, such as the use of CNTs with acid, are necessary to disperse the CNTs and improve the efficacy of removing heavy metals from wastewater. For this reason, several studies have enhanced the adsorption ability of CNTs by using a poly amido amine (PAMAM) dendrimer. This work revealed that the PAMAM/CNT nanocomposites were suitable for Ni^2+^, Zn^2+^, As^3+^, and Co^2+^ ion adsorption. The highest capacities for Ni^2+^, Co^2+^, Zn^2+^, and As^3+^ were 3900, 3800, 3650, and 3350 mg g^−1^, respectively [99]. Furthermore, thiol-functionalized multiwalled carbon nanotubes have been synthesized as successful adsorbents for Pb^2+^ ion removal, according to Qu et al. [100]. Since carbon-based polymer nanocomposites have greater properties than their counterparts or conventional carbonaceous materials, they have attracted much attention in recent studies. A polyamine/CNT adsorbent with a maximum adsorption capacity was synthesized as an ecologically friendly means of removing Cd^2+^ ions in research conducted by Adelabu et al. [101].

Although carbon nanotubes (CNTs) were determined to be the best adsorbent owing to their superior mechanical and chemical stability, a previous study did not report the recyclability of the adsorbent. These materials are difficult to separate from the solution and take a long time because they have no magnetic properties. Therefore, Nalini et al. explored the removal of arsenic by employing an adsorbent termed zero-valent iron (ZVI) on multiwalled carbon nanotubes (MWCNTs), which were doped with EDTA. EDTA functions as a chelating agent for arsenic removal and retains zero-valent iron. For As^2+^ and As^5+^, the composite maximum adsorption capacities at neutral pH were 111.1 ± 4.8 and 166.7 ± 5.8 mg g^−1^, respectively. Research on recycling has shown that composite materials may withstand up to five cycles without experiencing a substantial decrease in their capacity but take more time to separate the composite from the aqueous solution. Ultimately, without the need for any pretreatment, the adsorbent was effectively used to remove all inorganic arsenic from groundwater polluted with arsenic [102].

iii Graphene nanomaterials

Graphene is an alternative carbon-based nanomaterial that is becoming increasingly important for environmental regeneration. Although graphene is naturally hydrophobic, graphene oxide (GO) is an oxygen-containing complex that permits water contact. There are three different types of graphene materials: reduced graphene oxide (rGO), graphene oxide (GO), and pure graphene (pG). While rGO is the reduced form of GO, the oxidation of pG results in the formation of GO, which has several oxygen functional groups (hydroxyl, carbonyl, epoxide, and carboxyl). Among these materials, pG has been reported to be a structurally defect-free crystal [103]. Nonetheless, metal cation adsorption for wastewater treatment is favored by the high negative charge density and hydrophilic character of rGO and GO [104]. Amazing selectivity, affinity, removal capacity, and efficiency are displayed by functionalized pG, GO, and rGO in the treatment of wastewater polluted with metals [105]. Owing to their unique properties, which include radiation transmittance, high surface area, permeability, and thermal conductivity, these nanostructures have a wide range of applications, including advanced composites, biosensors, diffusion barriers, energy cells, membrane technology, protective overcoats, semiconductors, and supercapacitors. If graphene oxide nanosheets can be synthesized in the near future on a large scale and at a reasonable cost, they might be useful materials for removing heavy metal ion pollution. GO nanosheets were produced by Zhao et al. [106] for the adsorptive removal of Co^2+^ and Cd^2+^ ions. The adsorption capacities of Co^2+^ and Cd^2+^ ions were 68.2 and 106.3 mg g^−1^, respectively. The Cd^2+^ and Co^2+^ ion sorption process was significantly impacted by the many oxygen-containing functional groups on the graphene oxide nanosheet surfaces. Similarly, Arthi et al. used graphene oxide nanosheets as a type of nanosorbent material to remove Pd^2+^, Ni^2+^, and Cr^6+^ ions from pharmaceutical effluents. The findings demonstrated that GO efficiently eliminates all heavy metal ions at a concentration of 70 mg at a pH of 8 [107]. Highly ordered layered graphene oxide (GO) membranes were synthesized, and Ni^2+^, Cu^2+^, and Cd^2+^ ions were removed from aqueous solutions via these membranes as adsorbents. For Ni^2+^, Cu^2+^, and Cd^2+^ ions, the highest adsorption capacities of the GO membranes were approximately 62.3, 72.6, and 83.8 mg g^−1^, respectively [108]. In additional work, Yakoutet al. synthesized Zr-MnO_2_@reduced graphene oxide nanocomposite for efficient and simultaneous remediation of arsenates As(V) from environmental water samples [109]. The incorporation of Zr and MnO_2_ NPs plays a crucial role in enhancing the specific surface area and adsorption affinity of the RGO surface, resulting in an increased As(V) adsorption capacity. The removal efficiency remains almost constant (98.3%) after five consecutive sorption-adsorption cycles, which confirmed the reusability of the Zr-MnO_2_@RGO nanocomposite for arsenic treatment in real applicable scales. The results demonstrated that heavy metals may be effectively removed from water by using GO membranes as adsorbents.

### 2.2. Magnetic Nanomaterial (MNP)-Based Nanoadsorbent

A significant class of advanced nanomaterials are magnetic nanoparticles, which combine the benefits of nanotechnology with magnetic separation to provide superior adsorption capacity, affinity, and rate of adsorption compared with conventional adsorbing materials. The technique of magnetic separation is commonly applied in ecological challenges since the resulting MNPs have excellent reusability following their separation from solution by a straightforward magnetic process utilizing an external magnetic field [110]. As these magnetic nanoparticles decrease in size, their properties change significantly. New magnetic characteristics result from the single-domain structure of the magnetic material, which was formerly multidomain. Non-MNPs are less effective at decontaminating wastewater because of their smaller surface area and difficulty separating from the aqueous phase. On the other hand, MNPs are less poisonous and biocompatible, have a somewhat larger surface area, and are easily dispersed [110,111]. As a result, the MNPs used in wastewater treatment are more dependable, effective, and economical. Iron-based nanoparticles are thought to be among the greatest varieties of magnetic materials ever discovered. Maghemite (γ-Fe_2_O_3_), goethite (α-FeOOH), magnetite (Fe_3_O_4_), hydrous ferric oxides (HFOs), and hematite (α-Fe_2_O_3_) are different types of iron-based nanomaterials. The iron forms that are most often utilized are α-Fe_2_O_3_, Fe_3_O_4_, and γ-Fe_2_O_3_ because of their enormous surface area, magnetism, chemical stability, and less toxic effects. These three oxides differ greatly in their physical and magnetic properties according to changes in their crystal structures [112,113,114].

**Figure 6:**
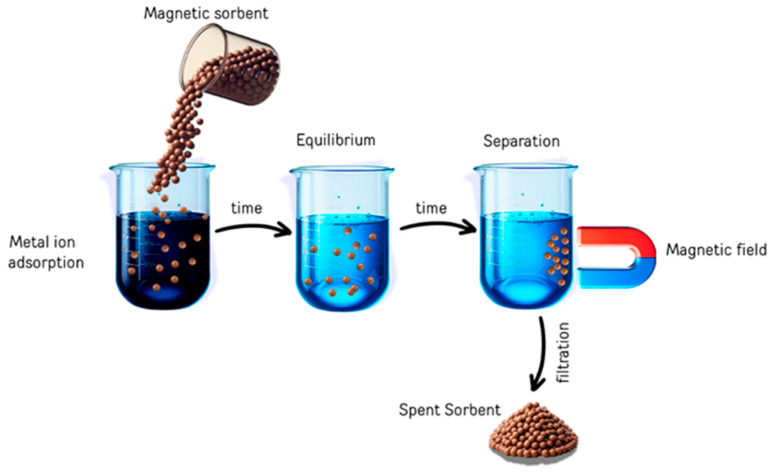
Wastewater treatment by adsorption of heavy metal ions onto magnetic nanoferrites used as adsorbents.

In the past several years, there has been a particular focus on the use of magnetized particles with micro- and nanoscales as adsorbents to eliminate metal ions from wastewater. Thus, the technology of magnetized particles has mostly played a role in resolving environmental issues [115,116]. Understanding the adsorption mechanism is essential since a successful adsorption process depends on the physicochemical interactions between the adsorbent and adsorbate. The adsorption process for heavy metal ions via magnetic ferrite adsorbents can be clearly described in Figure 6. Adsorption is apparently well recognized as a shift in mass, describing the process by which a material is transferred by physical or chemical interactions, or both, from a liquid phase to a solid surface. First, the van der Waals forces resulting from the altered electronic structure of the atom or molecule during adsorption are the foundation of physical adsorption or physisorption. Next, chemical adsorption/chemisorption essentially involves a chemical reaction on adsorbent surfaces [117]. In general, chemical adsorption is necessary to reach equilibrium with a longer contact time because of the strong chemical interaction between the adsorbent and the adsorbate, whereas physical adsorption necessitates a brief interval of contact time for the deposited heavy metal ions. On balance, heavy metal removal occurs on the solid surface of nanoparticles by adsorption, and equilibrium is attained by obtaining constant concentrations of the adsorbed heavy metals in wastewater [118,119]. Considering that chemical adsorption involves strong interactions, it can be confirmed that chemical adsorption results in greater adsorption capacities than physical adsorption. However, electrostatic interactions, hydrogen bonding, chemisorption, surface complexation, π–π interactions, and ion exchange are the primary causes of heavy metal adsorption onto spinel-structured ferrites and their composites [120]. Additionally, for ferrite adsorbents, pour filling is one of the most popular adsorption mechanisms because of the porous nature of the adsorbent and the kind of adsorbate.

Magnetic iron oxide nanoparticles are synthesized via a variety of techniques. When the diameters of the magnetite nanoparticles are less than 20 and 25 nm, the nanoparticles become super magnetic [121,122]. The saturation magnetization generally decreases with decreasing Fe_3_O_4_ nanoparticle size [123]. Moreover, magnetite iron oxide is readily oxidized to Fe_2_O_3_ or dissolved in an acidic solution; hence, it is important to achieve anaerobic conditions during synthesis to prevent oxidation [124]. Given this, it is clear that a variety of parameters influence the synthesis of magnetic nanoparticles, which, in turn, increases the method complexity. The diameters of the bare hematite, magnetite, and maghemite particles under various synthesis conditions are shown in Table 2.

Researchers have effectively removed heavy metal ions from various MNPs. This family has a large fraction of nanoscale iron oxide particles. These factors result in faster adsorption rates, easier separation, easier renewal, and greater adsorption capacities. S. Iconaru et al. [135] synthesized Fe_3_O_4_ nanoparticles and compared their efficacy with that of commercial magnetite to remove As^2+^ and Cu^2+^ ions. The results indicated that commercial magnetite had a lower adsorption capability than synthesized magnetite. The adsorptive elimination of Pb^2+^, Cu^2+^, Zn^2+^, and Mn^2+^ ions via Fe_3_O_4_ MNPs by Giraldo et al. was the subject of another investigation [136]. The Fe_3_O_4_ NPs were found to have a maximal adsorption ability for Pb^2+^ ions and a minimal adsorption capacity for Mn^2+^ ions. This is explained by the fact that the hydrated ionic radii of these heavy metal ions differ, causing variations in the electrostatic interactions between the negatively charged adsorption sites and the heavy metal cations. The coprecipitation method was used to create magnetite nanoparticles, which had a surface area of 94.43 m^2^ g^−1^, a pore size range of 2–60 nm, and a pore volume of 0.02–0.35 cm^3^ g^−1^. With maximum adsorption capacities of 42.75, 206.52, and 207.26 mg g^−1^ for As^3+^, Pb^2+^, and Cu^2+^ ions, respectively, the magnetic nanocomposite displayed good removal ability due to its large specific surface area, hydrophilic behavior, and functional moieties. Four consecutive adsorption–desorption cycles of the nanocomposite demonstrated the strong regeneration capability of the adsorbent [137]. Using porous Fe_3_O_4_ nanoparticles, Zawrah et al. investigated the simultaneous adsorption of Cd^2+^, Cu^2+^, and Pb^2+^ ions from water with removal efficiencies of 80, 84, and 86%, respectively. Adsorbent cycling was performed to test the reusability and stability of the material. The findings showed that the ionic radius of the cations had an effect on the adsorption efficiency trend, which was Pb^2+^ > Cu^2+^ > Zn^2+^ ions at pH 5.5, 6.5, and 6, respectively. The agglomeration and subsequent reduction in adsorption effectiveness occurred when the maximum suitable mass of adsorbent reached 200 mg. The adsorption process appears to have been well fitted to the Langmuir model. After three cycles, the adsorbent maintained approximately 90% of its initial sorption effectiveness with Pb^2+^, 40% with Cu^2+^, and 30% with Zn^2+^ ions [138]. In different studies, Zn^2+^, Cd^2+^, Pb^2+^, and Cu^2+^ ions from aqueous solutions were examined for their ability to adsorb via commercially prepared hematite nanoparticles (37.0 nm) in the following order: Pb^2+^ > Zn^2+^ > Cd^2+^ > Cu^2+^ ions. The desorption of metals from nano-hematite was pH dependent, with pH 4.0 > pH 6.0 > pH 8.0. The findings indicated that for all metals, more than 65% desorption was obtained at pH 4.0 in three 24 h cycles [139].

The particle size and structural surface area of magnetic adsorbents have a major impact on how well heavy metals are removed. The effectiveness of magnetic adsorbents in the removal of heavy metals and other pollutants from water and wastewater is confirmed by their large surface area, small nanoparticle size, and heterogeneous surface structure with high porosity [140]. Table 3 shows how alterations in the particle size and surface area of magnetic adsorbents impact the efficiency of heavy metal removal.

**Figure 7:**
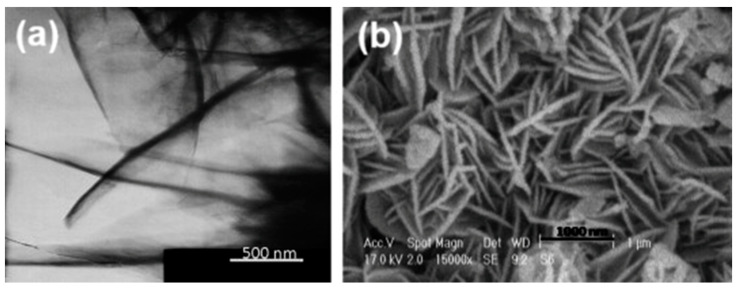
(**a**) SEM and (**b**) TEM images of the magnetite nanorods. Figure reproduced with permission of [149]. Copyright 2013, Elsevier.

The three types of nanostructures that have the largest specific area and the greatest ability to eliminate heavy metal ions from water are nanotubes, nanowires, and nanorods. In a study by Karami [149], magnetite nanorods (MNRs), which are new effective adsorbents for the removal of several heavy metal ions, such as Fe^2+^, Pb^2+^, Zn^2+^, Ni^2+^, Cd^2+^, and Cu^2+^ ions, from aqueous solutions, were synthesized via an electrochemical method. SEM and TEM analyses revealed that the synthesized sample consisted of magnetite nanorods (MNRs) with an average diameter of 60 nm and an average length of 1000 nm, as illustrated in Figure 7. The magnetite nanoadsorbents exhibited maximum adsorption capacities of 79.10, 88.39, 95.42, 107.27, 112.86, and 127.01 mg g^−1^ for the Cu^2+^, Cd^2+^, Ni^2+^, Zn^2+^, Pb^2+^, and Fe^2+^ ions, respectively. The experimental results indicate that the synthesized magnetite nanorods are effective at quantitatively extracting heavy metal ions from water.

**Figure 8:**
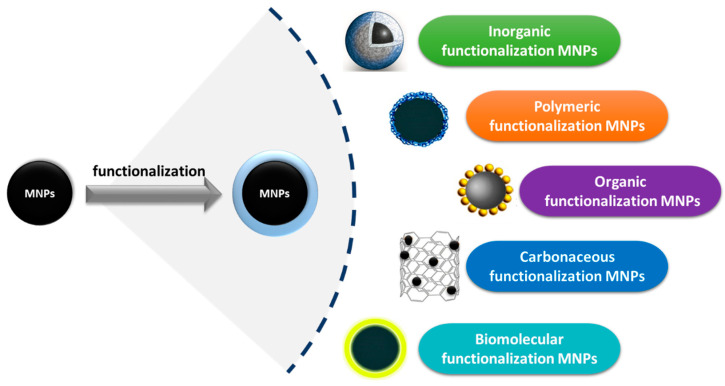
Schematic illustration of the surface functionalization of magnetic nanoparticles.

Bare MNPs often aggregate quickly in aqueous media, which limits their practical use. In addition to the notable advancements in MNP synthesis, a crucial concern is preserving MNP stability by avoiding precipitation and agglomeration. Better selectivity towards a certain metal ion can be achieved by functionalizing bare MNPs with various moieties, hence overcoming these limitations. MNP surface modification also increases their adsorption ability and resistance to agglomeration. Functionalization provides support for chemical binding and complex formation in addition to van der Waals and electrostatic interactions, which regulate metal ion adsorption on the adsorbent surface [150,151]. The charge on the nanoadsorbent surface is altered by functionalization with charged functional groups, which also increases the electrostatic interactions between the metal ions and the adsorbent surface. The overall size and surface area of MNPs, which are essential for the improved and selective adsorption of heavy metal ions from wastewater, are determined by the type of surface functionalization and the geometric arrangement of the coating molecules. Numerous studies have examined the functionalization of MNPs with a range of materials, as illustrated in Figure 8. These materials include inorganic, organic, carbonaceous, and polymeric materials.

#### 2.2.1. Inorganic-Functionalized MNPs

**Figure 9:**
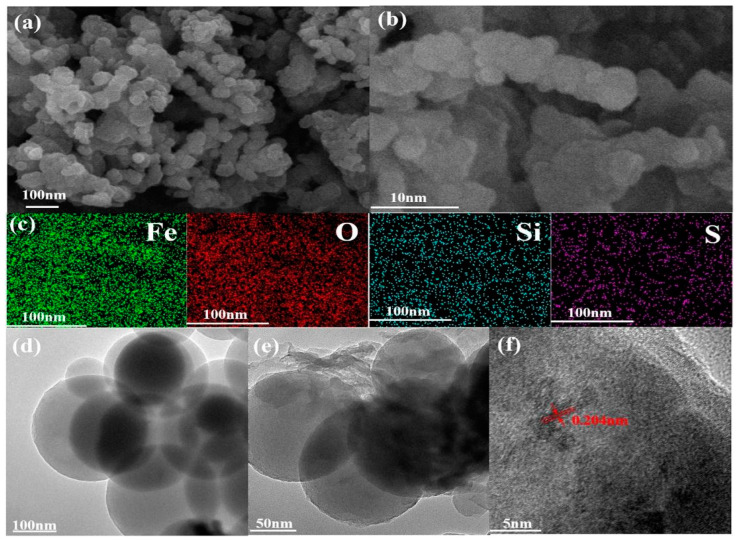
Characterization of NZVI-SH-HMS: SEM (**a**,**b**), EDS mapping (**c**), TEM (**d**,**e**), and HRTEM (**f**) images. Figure reproduced with permission of [153]. Copyright 2021, Elsevier.

In metal removal applications, inorganic chemicals such as EDTA and silica have also been utilized for MNP functionalization. In light of this, Ghaemi et al. [152] modified various wt.% Fe_3_O_4_ NPs with silica, metformin, and amine. The maximum removal of Cu^2+^ ions (approximately 92%) was shown for the Fe_3_O_4_ MNPs coated with metformin-modified silica (0.1 wt.%), owing to the high affinity of Cu^2+^ adsorption. In another study, functionalized magnetic mesoporous silica (NZVI-SH-HMS), which has thiol groups and nanometer zero-valent iron immobilized on its surface, was effectively used to eliminate Pb^2+^ and Cd^2+^ ions from aqueous solutions. This material was synthesized at room temperature via sol–gel and wet impregnation techniques. SEM, TEM, and FTIR analyses demonstrated that the NZVI and thiol groups were uniformly and stably distributed across the HMS surface. The regular spherical shape with mesoporous channels is displayed in Figure 9a,b. Concurrently, the EDS mapping spectrum (Figure 9c) demonstrated a homogeneous distribution of Fe and S on the NZVI-SH-HMS surface, indicating that NZVI and thiol were successfully loaded. In the TEM images, Figure 9d,e, the clean and single morphology of NZVI-SH-HMS also revealed a normal spherical shape with a characteristic wormhole mesoporous structure, which further suggested the uniform distribution of NZVI and thiols. The HRTEM image (Figure 9f) revealed a crystalline lattice fringe of 0.2 nm, which was attributed to the (110) diffraction plane of iron. The composite has good porous characteristics, according to N_2_ adsorption–desorption isotherms, with a large specific surface area (312.84 m^2^/g) and the right pore size (2.56 nm). The superparamagnetic nature of the material was verified by XRD and XPS data. Furthermore, NZVI-SH-HMS demonstrated outstanding sorption recyclability and adsorption capacities in a variety of simulated wastewater scenarios, indicating the potential of this material for eliminating heavy metal ions in real-world aquatic environments. These findings revealed that the adsorption performance of functionalized magnetic mesoporous silica materials was significantly enhanced by the combined actions of NZVI and thiols [153].

Magnetic graphene oxide (Fe_3_O_4_-GO@SiO_2_) composites covered with silica were created by Suo et al. via a simple method. To extract Ag^+^, Cd^2+^, Co^2+^, Cr^3+^, Cu^2+^, Ni^2+^, and Pb^2+^ ions from ambient water samples at detectable levels, the nanocomposite was characterized and used. Four ambient water samples had metal recoveries ranging from 83% to 109%, with relative standard deviations (RSDs) less than 7%. Additionally, the adsorption capacities of Fe_3_O_4_-GO@SiO_2_ for Cd^2+^, Co^2+^, Ag^+^, Cu^2+^, Pb^2+^, Cr^3+^, and Ni^2+^ ions were 100.81, 116.35, 141.09, 149.59, 168.55, 182.98, and 226.08 mg g^−1^, respectively. Ultimately, the devised approach was effectively used to detect target metals at low levels in ambient water samples simultaneously and sensitively [154]. Additionally, according to Abu Taleb et al., a unique multifunctional SiO_2_/CuFe_2_O_4_/polyaniline composite was synthesized by interacting silica (SiO_2_), copper iron oxide (CuFe_2_O_4_), and polyaniline (PANI) as the starting components. The sensitized composite was characterized and used for wastewater solutions to remove Cu^2+^, Fe^2+^, and Mn^2+^ ions with high capacities of 285.71, 416.67, and 454.55 mg g^−1^, respectively. The binding of metal ions onto the composite was caused primarily by electrostatic attractions and chelation. The composite adsorption active sites and hydrated ionic radii primarily controlled the selectivity of the examined ions. After four successive regeneration cycles, SiO_2_/CuFe_2_O_4_/PANI may be reused with ease, but the effectiveness of metal removal decreases somewhat (by approximately 2–3%) [155]. In a different study, Fe_3_O_4_-SiO_2_ magnetic composites were effectively synthesized by Xu et al. [156], and they were then used to remove Pb^2+^ ions. The produced nanosorbents had many Pb^2+^ adsorption sites and were uniform in size. Their strong paramagnetic nature also made extraction easier with an external magnet. The Pb^2+^ ion adsorption ability reached a maximum as the temperature increased. Similarly, Xu et al. [157] investigated the removal of metal ions with adsorption capabilities in the following order: Fe^3+^ > Cu^2+^ > Cr^3+^ ions using MNPs modified with polymers and silica (MSNP–CAAQ). The complexation process is regulated by the electrical properties of the metal ion and the ligand, which produce an observable sequence. Jamasbi et al. [158] employed MNPs modified with 1,4-butane sultone (–SO_3_H) groups covered in silica in another scientific study. Even at extremely low concentrations, MNPs coated with silica were shown to be very effective at eliminating Pd^2+^ ions. Currently, researchers often employ mixed metal oxide composites as adsorbents to extract heavy metal ions from wastewater. To remove As^5+^ ions from aqueous solutions, Tran et al. [159] synthesized a Fe_2_O_3_/TiO_2_ composite by treating titanium slag with a low-concentration sulfuric acid solution (20%). Batch adsorption was employed to investigate the removal efficiency of the Fe_2_O_3_/TiO_2_ adsorbent toward As^5+^ ions. The adsorption of As^5+^ ions on Fe_2_O_3_/TiO_2_ indicates a multilayer adsorption process with an adsorption capacity of 68.26 mg g^−1^. The pseudo-second-order adsorption behavior of the Fe_2_O_3_/TiO_2_ composite for As^5+^ ions was investigated via adsorption kinetics. The produced Fe_2_O_3_/TiO_2_ adsorbent has a high adsorption capacity and can be inexpensive, making it a useful adsorbent for removing As^5+^ ions from polluted water sources. The methodology employed in this study is thought to be a viable path toward developing an effective adsorbent for water purification.

As nanosorbents, magnetic nanocomposites of various metal oxides have also been studied. Kim et al. [160] created a Fe_3_O_4_/MnO_2_ nanocomposite in the form of a flower. For the Cd^2+^, Cu^2+^, Pb^2+^, and Zn^2+^ ions, the adsorbent demonstrated a much greater adsorption performance than the Fe_3_O_4_ NPs. Metal ferrite nanoparticles (NPs), which combine ferrite molecules with any metal to achieve magnetic separation, constitute another significant class of magnetic nanomaterials. Metal ferrite is often written as M(Fe_x_O_y_), where M represents any metal that forms divalent bonds. Several investigations have generated Mn-Zn ferrite (Mn_0.67_Zn_0.33_Fe_2_O_4_) NPs [161] with the purpose of removing heavy metal ions such as As^5+^, Cd^2+^, and Pb^2+^ ions with good removal efficiency. MnFe_2_O_4_ and CoFe_2_O_4_ spinel ferrite nanoparticles were produced by Asadi et al. [162] in another work. Zn^2+^ ions were extracted from an aqueous solution via the produced nanoparticles, which had 454.5 and 384.6 mg g^−1^ adsorption capacities.

It is also possible to functionalize magnetic nanoparticles via carbonate-based adsorbents, which is another family of materials. This inexpensive, nontoxic substance with a high solubility product effectively increases the metal ion adsorption efficiency of adsorbents by encouraging precipitation interactions between calcium carbonate and heavy metal ions. However, several drawbacks limit the use of CaCO_3_ in wastewater purification, including low efficiency, sludge production, and challenging separation. CaCO_3_ and MNPs may be employed to improve wastewater separation and increase the adsorption capacity to overcome these technical obstacles. In this context, Wang et al. [163] prepared a magnetic composite from iron oxide and CaCO_3_ via a hydrothermal technique. This composite removed Cd^2+^ and Pb^2+^ ions with maximum adsorption capacities 821 and 1179 mg g^−1^ faster than any other conventional adsorbent, according to earlier findings.

#### 2.2.2. Organic-Functionalized MNPs 

**Figure 10:**
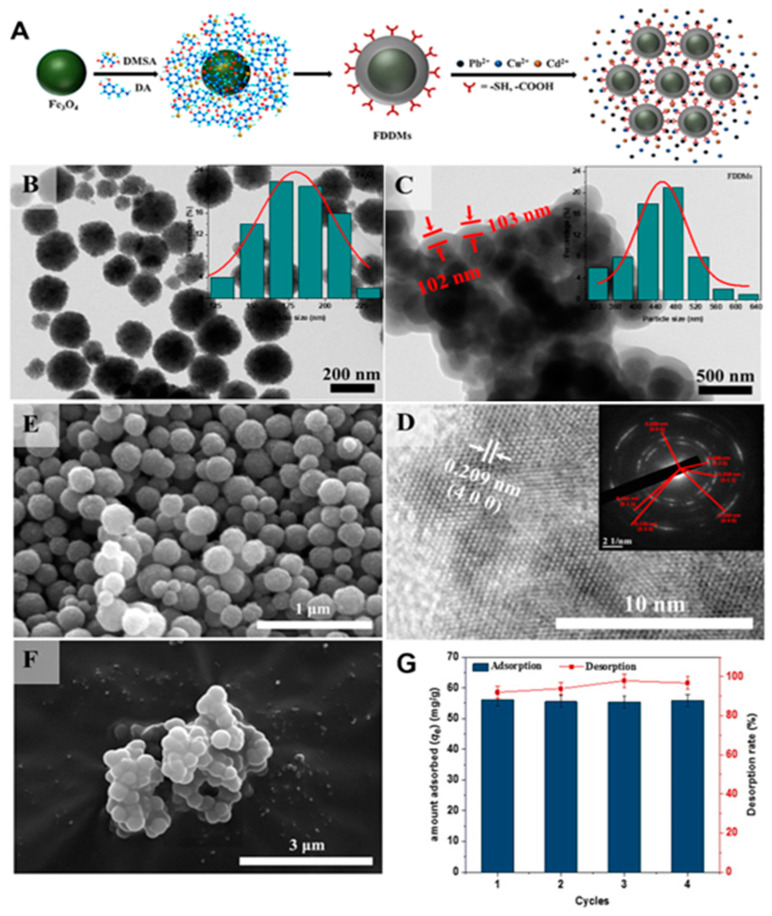
Fe_3_O_4_@DA–DMSA magnetic nanoparticle synthesis and the method of eliminating heavy metal ions (**A**), TEM images and size distribution curves of the Fe_3_O_4_ and nanocomposite, respectively (**B**,**C**), HRTEM image of Fe_3_O_4_ (**D**), SEM images of the Fe_3_O_4_ nanocomposite, respectively (**E**,**F**), and Pb^2+^ ion adsorption capacity and desorption rate by the adsorbent following various cycles of absorption and desorption (**G**). Figure reproduced with permission of [164]. Copyright 2023, Elsevier.

**Figure 11:**
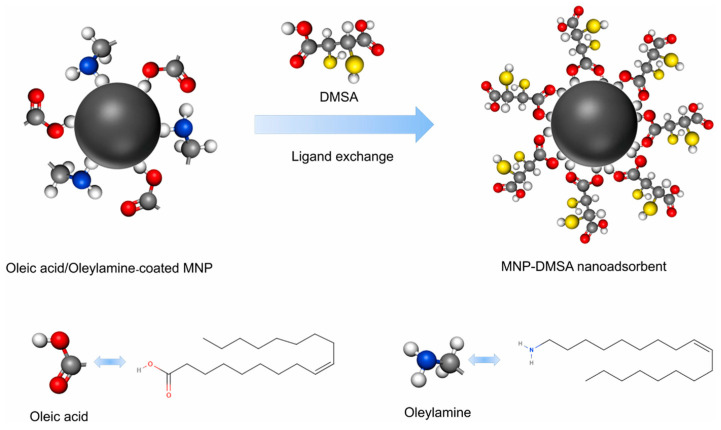
The ligand exchange process used to functionalize MNPs with DMSA involved DMSA ligands replacing the oleic acid and oleylamine surfactant groups. Figure reproduced with permission of [165]. Copyright 2023, Elsevier.

The many functional groups that organic molecules provide for complexation with ions of heavy metals greatly increase the adsorption capacity of MNPs. Lei et al. [164] synthesized a Fe_3_O_4_ magnetic core via a solvothermal method to eliminate heavy metal ions. They then functionalized the magnetic core surface with DA and DMSA, resulting in a bifunctional core–shell structure, as shown in Figure 10A. These nanocomposites were then characterized via various spectroscopic techniques. TEM and SEM images of the Fe_3_O_4_ and nanocomposite were captured to obtain more precise information on the particle size and shape of the adsorbent (Figure 10B–F). The produced Fe_3_O_4_ nanoparticles showed excellent dispersion in the TEM images (Figure 10B). A TEM image of the nanocomposite is shown in Figure 10C. The nanocomposite exhibited a core–shell structure, where the Fe_3_O_4_ particles appeared dark as the core, and a uniform light-colored modification layer (100 nm thickness) consisting of DA-DMSA was observed coating the Fe_3_O_4_ surface. Figure 10D shows high-resolution TEM (HRTEM), which revealed a uniform and full lattice structure of an individual Fe_3_O_4_, with a plane lattice spacing of 0.209 nm, which corresponds to the (400) plane in Fe_3_O_4_. The Fe_3_O_4_ surface was coated with a modified layer, as shown in the SEM images (Figure 10E,F). Moreover, upon comparing the EDS spectra of Fe_3_O_4_ and the nanocomposite, the presence of S and N in the nanocomposite indicates successful coating of the DA-DMSA layer on the Fe_3_O_4_ surface. Furthermore, the TEM and SEM images of the nanocomposite revealed aggregation of the adsorbent, which is consistent with the adherence of the coating and the magnetic attraction of the magnetic core. The ability of the as-prepared magnetic nanoparticles to adsorb Cd^2+^, Cu^2+^, and Pb^2+^ ions was investigated. According to the findings, Cd^2+^, Cu^2+^, and Pb^2+^ ions had maximal adsorption capacities of 49.46, 63.01, and 187.62 mg g^−1^, respectively. Compared with the most widely published Fe_3_O_4_ MNP-related adsorbents, the nanocomposite had the highest capability for Pb^2+^ ion removal. Future water treatment initiatives are anticipated to use nanocomposites as potential adsorbents for the treatment of wastewater containing Pb^2+^ ions. In another study, monodispersed Fe_3_O_4_ MNPs were effectively functionalized by V.P. Kothavale et al. [165] via DMSA via the ligand exchange process (Figure 11). The removal of Cd^2+^, Pb^2+^, and Ni^2+^ ions from aqueous solutions was accomplished concurrently through the use of MNP-DMSA as an efficient nanoadsorbent. The MNPs exhibited a spherical form and a pure magnetite phase, with an average particle size of 8.24 ± 1 nm. The presence of carboxyl and thiol functional groups on MNP-DMSA was shown by FTIR and XPS investigations. With an Ms value of 62.8 emu/g, the MNPs were superparamagnetic at room temperature. Surface areas of 123 and 137 m^2^/g for MNPs and MNP-DMSA, respectively, were found via analysis using BET theory. Zeta potential tests revealed that MNP-DMSA had a greater negative surface charge than bare MNPs, which contributed to its hydrophilic character and superior colloidal stability. The simultaneous removal of Cd^2+^, Ni^2+^, and Pb^2+^ ions from the ternary system resulted in maximal adsorption capacities of 27.18, 53.9, and 64.5 mg g^−1^, respectively. The adsorption capacities for Cd^2+^, Ni^2+^, and Pb^2+^ ions were increased to 75.48, 102.73, and 116.54 mg g^−1^, respectively, in single systems. Five adsorption–desorption cycles revealed the high reusability of the MNP-DMSA nanoadsorbent. These findings indicate that the MNP-DMSA nanoadsorbent has great potential for the concurrent elimination of many metal ions from aqueous solutions.

Additionally, amino-functionalized magnetic nanoadsorbents (MNP-NH_2_) efficiently removed heavy metal ions. Mabes Raj et al. [166] reported that amino-functionalized γ-Fe_2_O_3_ NPs effectively removed Pb^2+^ and Hg^2+^ ions from aqueous solutions at pH values of 4 and 7, with adsorption capacities of 85.6 and 83.6 mg g^−1^, respectively. Similarly, Gao et al. [167] functionalized mesoporous superparamagnetic Fe_3_O_4_ NPs by using triethylenetetramine as a chelating ligand. With an 85% removal efficiency, the removal of copper from wastewater has been demonstrated via these functionalized MNPs. Shen et al. [168] investigated the mechanism of Cr^6+^ ion adsorption on the surface of nanosized magnetic polymers from tetraethylene pentamine. A variety of core–shell Fe_3_O_4_ NPs functionalized with amino groups (NH_2_-NMPs) were synthesized by Shen et al. [169] for the adsorption of Cu^2+^ and Cr^6+^ ions in both solo- and coexisting metal ion systems. The adsorption process for both Cr^6+^ and Cu^2+^ ions was shown to be highly dependent on the pH of the medium. The primary reason for this was that the adsorption process relied heavily on electrostatic interactions. The maximum adsorption capabilities were observed at pH values ranging from 2 to 4. The lower availability of -NH_2_ binding sites at pH values below 2 is caused by the protonation of –NH_2_ to –NH_3_^+^, which decreases the adsorption effectiveness. Cu^2+^ ion adsorption increases at pH values greater than 2 due to the increased availability of -NH_2_ binding sites. On the other hand, Cu^2+^ precipitates at pH values higher than 4. Furthermore, in systems with relatively high metal ion concentrations and relatively low pH values, the adsorption process was competitive. Higher pH values and lower concentrations had little effect on the adsorption efficiency of the coexisting systems. Bobik et al. [170] synthesized polysodium acrylate (PSA)-coated Fe_3_O_4_ NPs, which were shown to be both economical and ecologically benign for the sorption of Ni^2+^, Cd^2+^, Zn^2+^, Pb^2+^, and Cu^2+^ ions. The results demonstrated that the Fe_3_O_4_/(PSA) nanocomposite was more stable and had a greater capacity for adsorption.

**Figure 12:**
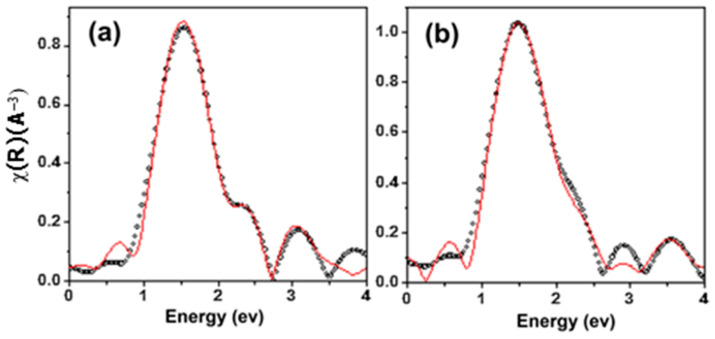
Fourier–transformed EXAFS spectra of (**a**) liquid and (**b**) solid at U L3 edge. The experimental spectra are represented by scatter points and the theoretical fit is represented by a solid line. Figure reproduced with permission of [171]. Copyright 2020, Elsevier.

In another study for removal of uranium (IV) from wastewater according to purification of tap water, drinking water, and sea water, Singhal et al. [171] synthesized phosphoramide-functionalized Fe_3_O_4_ nanoparticles (NPs) for uranium extraction from different environmental matrices. The results indicate that the maximum adsorption capacity of the adsorbent reached 95.2 mg g^−1^. Studies on uranium L_3_-edge EXAFS have been conducted in phosphoramide-functionalized Fe_3_O_4_ NPs (after adsorption) and in liquid (before sorption). The best fit was obtained with a structural model having three coordination shell of oxygen atoms in both samples. The first shell in liquid was made up of two axial oxygen (O_ax_) atoms at a distance of 1.82 ± 0.01 Å, while the second shell was made up of two equatorial oxygen (O_eq_) atoms at a distance of 2.30 ± 0.01 Å. Three oxygen (O_eq_) atoms at a distance of 2.49 ± 0.01 Å were found in the third shell. Two axial oxygen (O_ax_) atoms at 1.79 ± 0.01 Å, two equatorial oxygen atoms at (O_eq_.) at 2.32 ± 0.01 Å, and three equatorial oxygen atoms at (O_eq_.) 2.42 ± 0.02 Å were displayed by the fitting parameter following sorption at solid. Additionally, the fourth shell or the second coordination sphere was also fitted. Three more oxygen atoms were observed in the liquid at a distance of 3.49 ± 0.04 Å. These might be the outcome of interactions between coordinated water molecules in liquid and hydrogen bonds. The fourth shell in a solid was made up of three phosphorus atoms spaced 3.92 ± 0.02 Å apart. This demonstrates that the three oxygen atoms linked to uranium (VI) at the solid surface come from the phosphine oxide functional group that is present. The fitted spectra are shown in Figure 12. Based on the EXAFS studies, the majority of uranium (VI) present in the solution at a concentration of 1 ppm occurs as UO_2_ (H_2_O)_n_ L_m_, where L may be CO_3_^2−^ or OH. The parameters obtained from the fitting of EXAFS spectra of solid U@ Fe_3_O_4_ suggest that the P=O group on the surface of Fe_3_O_4_ binds to the uranyl center by replacing the water molecule, making the complex UO_2_Lm.3 (P=O) (where P=O is the surface functional groups, and L can be OH or CO_3_^2−^). Based on the good agreement between the acquired U-P distance of 3.92 Å and the total of the U=O bond distance (obtained from EXAFS) of 2.42 Å and P=O bond length (1.50 Å), a proposal was made about the binding mechanism of uranium with the synthesized sorbent.

#### 2.2.3. Carbonaceous Material-Functionalized MNPs

**Figure 13:**
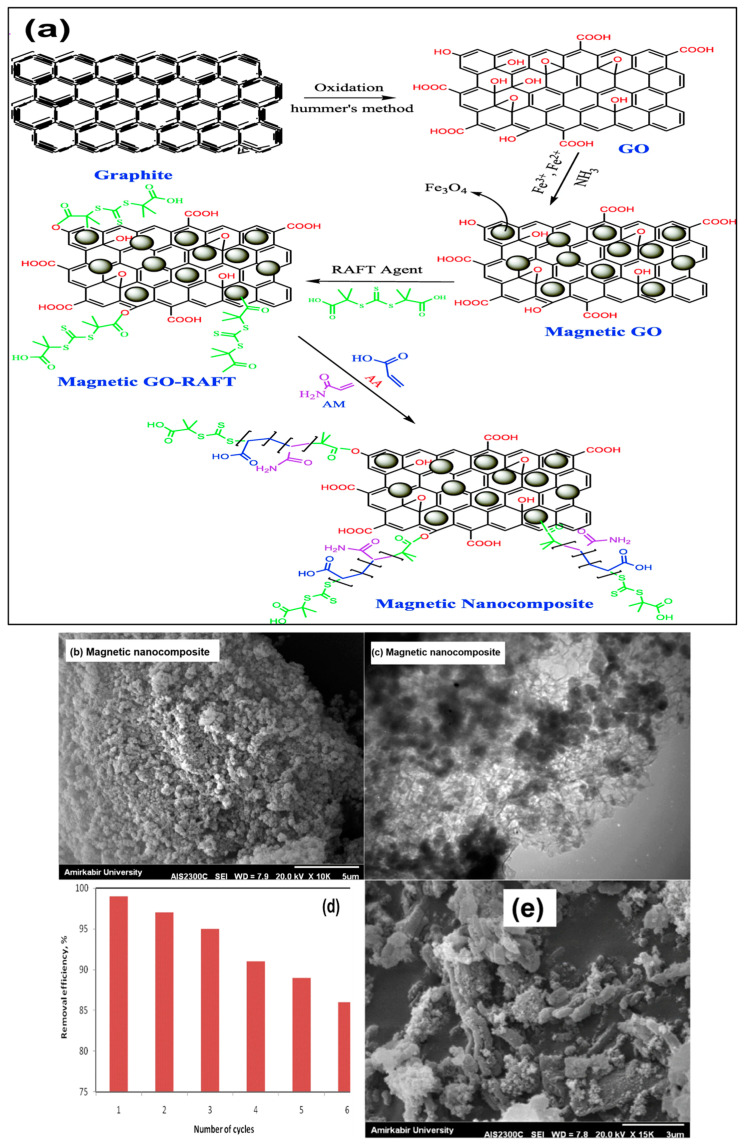
(**a**) Schematic illustration of the fabrication of magnetic nanocomposite (MNAs) adsorbents (MNAs); (**b**,**c**) SEM and TEM images of the synthesized magnetic nanocomposite, respectively; and (**d**) the reusability of (MNAs) during five cycles of adsorption–desorption processes and (**e**) SEM images of (MNAs) after adsorption. Figure reproduced with permission of [172]. Copyright 2019, Springer.

**Figure 14:**
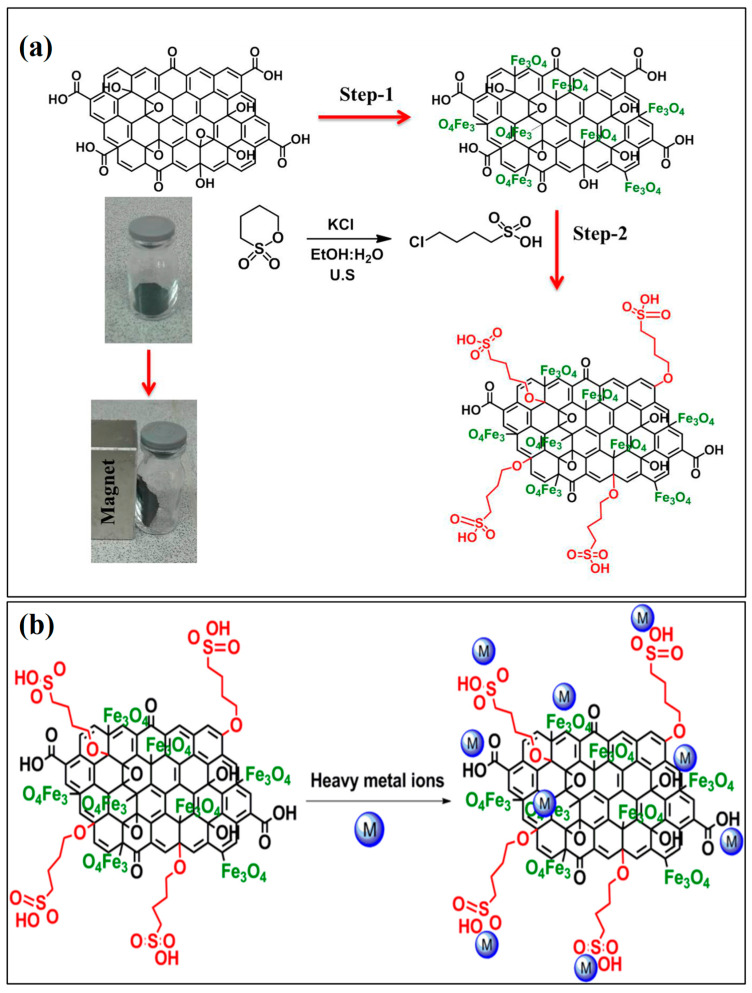
(**a**) The synthesis methods and structure of Fe_3_O_4_ NPs@AC@C_4_H_8_SO_3_H composites and (**b**) the proposed mechanism for metal ion adsorption on the surface of Fe_3_O_4_ NPs@AC@C_4_H_8_SO_3_H composites. Figure reproduced with permission of [176]. Copyright 2019, Elsevier.

Additionally, one efficient way to increase the adsorption effectiveness of nanoadsorbents is to functionalize (MNPs) with carbonaceous materials such as activated carbon and graphene oxide (GO). For example, Hosseinzadeh et al. [172] used surface reversible addition–fragmentation chain transfer copolymerization of acrylic monomers to synthesize a magnetic nanocomposite based on GO, as shown in Figure 13a, for the sorption of Hg^2+^ ions. In the SEM image of the magnetic nanocomposite, as shown in Figure 13b, a uniform roughness was obtained at the surface because of the linkage of the polymeric chains onto the magnetic GO. Conclusively, the SEM results indicate an acceptable improvement in the porosity of the modified magnetic nanocomposite. This porous structure can be used as a good candidate site for the removal of various molecules and ions. As seen in the TEM images in Figure 13c, the magnetic GO converts to a rough surface because of the grafting reactions of acrylic monomers on the GO surface. High selectivity and adsorption efficiency (MNA) for Hg^2+^ ions were shown by the findings in the presence of competing ions. Additionally, Hg^2+^ ion removal followed a Langmuir model, with high Hg^2+^ ion adsorption of 389 mg g^−1^. After the five-regeneration cycle, the MNA was found to be a suitable material for reuse and retained 86% of its initial capability for adsorbing mercury. Moreover, it should be noted that the MNA substrate maintains its initial shape after mercury adsorption and desorption processes (Figure 13d), which confirms its high cycling stability. Finally, the experimental results indicated that the Hg^2+^ ion adsorption capacity was highly influenced by the temperature, ion concentration, and pH of the solution. These findings suggest that MNAs with strong adsorption capabilities may prove to be highly advantageous adsorbents for the selective recovery of ions throughout the wastewater treatment procedure. H. Su et al. synthesized iron oxide–GO nanocomposite adsorbents (FeO_x_-GO-36, FeO_x_-GO-60, and FeO_x_-GO-80) with varying iron oxide contents in addition to an iron oxide control sample to remove arsenic. Compared with those of the GO and iron oxide control samples, the nanocomposite adsorbents exhibited noticeably greater arsenic adsorption capabilities. Owing to the creation of more accessible active sites and increased surface area, increased iron oxide concentrations are associated with increased arsenic adsorption capabilities. Among the numerous iron oxide–GO/rGO composite adsorbents reported to date, FeOx-GO-80 has the greatest adsorption value, with values of 113 and 147 mg g^−1^ for As^5+^ and As^3+^ ions, respectively. FeO_x_–GO nanocomposites have a great deal of potential for removing arsenic from practical water treatment, especially for drinking water purification, because of the easy synthesis of the nanocomposites and the inexpensive cost of GO, which can be made from abundant natural graphite [173]. Another study used a precipitation process to produce a new magnetic activated carbon composite (AC/NiF), which was then used to retain Cu^2+^ and Zn^2+^ ions in aqueous solutions. The magnetic composite was found to have a retention ability towards both metal ions that was significantly greater than that of activated carbon (AC) [174]. This confirms the magnetic composite’s status as a novel and promising material, which presents new opportunities for achieving the necessary adsorption and effective magnetic separation. Ghasemi et al. [175] synthesized highly efficient Fe_3_O_4_ NPs functionalized with EDTA for the adsorption of Ag^+^, Hg^2+^, Mn^2+^, Zn^2+^, Pb^2+^, and Cd^2+^ ions, with adsorption capacities and efficiencies ranging from 71 to 169 mg g^−1^ and 96 to 104%, respectively. In accordance with Figure 14a, V. Nejadshafiee et al. [176] created a unique magnetic bioadsorbent by immobilizing 1,4-butane sultone and loading Fe_3_O_4_ NPs onto the surface of activated carbon. By utilizing pistachio shells as a biogenic, bioresource carbon material, activated carbon was produced. The morphology, structure, and magnetic properties of the prepared adsorbent were studied. Under ultrasound assistance, the maximal adsorption rates of Cd^2+^, Pb^2+^, and As^3+^ ions were 119.04, 147.05, and 151.51 mg g^−1^, respectively. This adsorbent has significant adsorption capacity in addition to its benefits, including low cost, simple separation, recyclability, and green synthesis. Figure 14b illustrates the suggested mechanism for the adsorption of heavy metal ions by the composites. The heavy metal ions form a complex with the SO_3_H and OH^-^ groups present on the surface of the composites. The loading of heavy metal ions onto the composites was verified by further analysis of the FT-IR spectra. As a result of the adsorption of heavy metal ions, the OH hydroxyl, carboxyl, and carbonyl absorption bands of the composites significantly changed. This indicates that the functional groups interacting with the heavy metal ions are responsible for the observed changes in the number of absorption bonds.

A novel adsorbent with exceptional magnetic characteristics, MnFe_2_O_4_/multiwalled carbon nanotubes (MMWCNTs), was synthesized by Zhao et al. and characterized extensively. The problem of removing the nanoadsorbent from the liquid medium was solved by adding MnFe_2_O_4_. The findings of a batch adsorption experiment revealed that a pH of 6–8 was sufficiently neutral for successful adsorption. At 308 K, the greatest adsorption capacity was 46.41 mg g^−1^. The endothermic nature of MMWCNT adsorption was demonstrated by adsorption thermodynamics, and the adsorption process benefited from increasing temperature. Moreover, the many functional group surfaces of the MMWCNTs improved the adsorption efficiency. Compared with that of the remodified MWCNTs, the removal efficiency of the adsorbent for Cu^2+^ ions increased almost 10-fold, from 3.4% to 34.8%. These findings showed that MMWCNTs are effective and appropriate adsorbents for adsorbing Cu^2+^ ions in natural water bodies [177].

#### 2.2.4. Polymer-Functionalized MNPs

**Figure 15:**
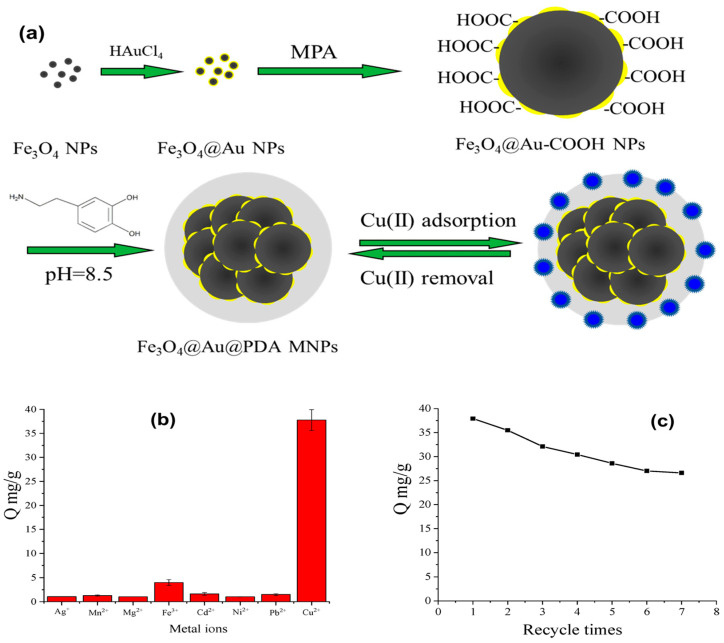
(**a**) Synthesis route of magnetic nanocomposites, (**b**) selectivity adsorption of MNPs toward metal ions, and (**c**) the effect of regenerative times on the adsorption capacity [178].

Polymers have also been employed to alter the surfaces of magnetic nanoparticles to improve their adsorption capacity. The structural modification of NPs with polymeric support has various benefits, including high mechanical strength, biocompatibility, and chemical stability. Numerous studies have demonstrated how to combine polymers with MNPs to enhance the mechanical and thermal characteristics of the polymer. For example, it was possible to create unique magnetic Fe_3_O_4_@Au@PDA nanocomposites [178] that efficiently adsorbed Cu^2+^ ions. The as-prepared MNPs can reach saturation after two hours of adsorption in a neutral environment, according to the results. The adsorption efficiency of the MNPs was highly dependent on the temperature, solution pH, and starting Cu^2+^ ion concentration. The excellent adsorption behaviors were dominated by the rich catechol groups of polydopamine. Furthermore, MNPs may be regularly used and readily desorbed at temperatures higher than 60 °C. Moreover, Figure 15 illustrates that the produced MNPs exhibit high selectivity for Cu^2+^ ion removal. MNPs are effective and ecologically benign adsorbents for the selective elimination of Cu^2+^ ions from aqueous solutions, according to the results. The synthesis of polymer magnetic nanocomposites using polyacrylonitrile (PAN) was the subject of a different investigation by Dogari et al. [179]. The authors demonstrated the efficacy of these MNCs in decontaminating water that included Pb^2+^ and Cd^2+^ ions. Additionally, Co^2+^ and Ni^2+^ ions were successfully adsorbed via chitosan as a polymer in another study by Sharifi et al. [180] on polymer–iron oxide MNPs. Iron oxide nanoparticle beads have a high adsorption capacity at normal pH and quick equilibrium periods, making them effective adsorbents. In a similar study, Huang et al. [181] reported the use of magnetic chitosan composites in another investigation. According to the experimental data, the composite tended to effectively remove Pb^2+^ ions. Furthermore, Tsague et al. [182] demonstrated how to apply a novel magnetic nanoadsorbent for the adsorption of Pb^2+^ ions by functionalizing Fe_3_O_4_NPs with polyethyleneimine. The composites effectively took up Pb^2+^ from aqueous media.

Additionally, Table 4 provides an overview of the experimental conditions for metal ion removal using the previously mentioned different types of nanoadsorbents.

### 2.3. Separation of Magnetic Nanoadsorbent from the Reaction System

Adsorbent reusability is an important consideration when assessing their feasibility in real-world applications, as an effective repeatable adsorbent supply lowers the total cost of the adsorption technique. After organic and inorganic impurities have been removed from aqueous solutions, an external magnetic field can flow through glass and plastic materials to separate the magnetic nanoparticles used without the need for filtering. Afterward, several desorption procedures can be used to regenerate new nanomaterials. HCl and HNO_3_ solutions have also been utilized as desorbing agents for the regeneration of metal-loaded adsorbents. These encouraging aspects suggest that nanoparticles are reasonably priced adsorbents that have the potential to be recycled. Researchers have published a number of studies outlining various techniques for removing adsorbents from chemical systems and recycling nanoadsorbents. Zeng et al. [230] removed Pb^2+^ ions from an MNGH nanoadsorbent by using a 0.5 M HNO_3_ solution. The Pb^2+^ ion adsorption ability decreases somewhat with time as a result of the number of reuse cycles. It maintained approximately 87% of its initial adsorption capacity even after eight cycles of reusability. As a result, MNGH demonstrated remarkable reusability in the elimination of heavy metal ions. A study carried out by Wu et al. [231] revealed that in a 10-cycle adsorption–desorption test, the arsenic removal efficiency of GCFF began to decrease only after the ninth regeneration cycle.

**Figure 16:**
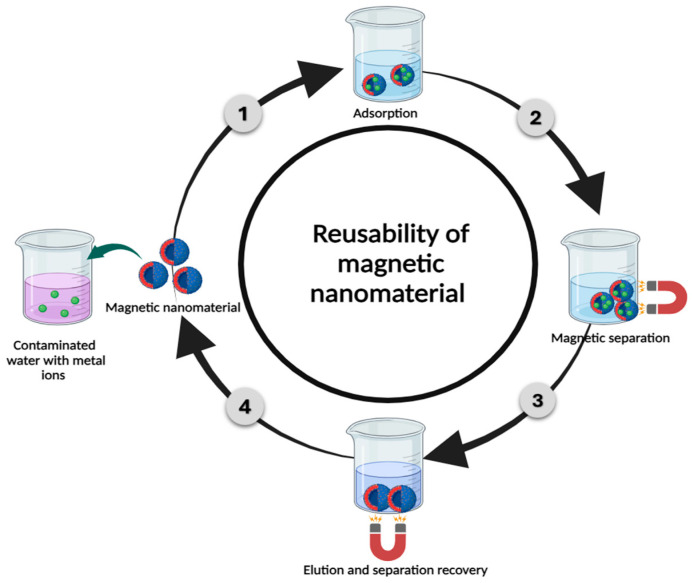
Schematic diagram illustrating the contaminant adsorption, desorption, recovery, and reuse process for the application of magnetic nanomaterials in aqueous solution.

Another scientific study used a diluted HNO_3_ solution as the desorbent to assess the regeneration ability of HNC-3. After five cycles, the removal efficiency for Pb^2+^ ions remained consistent, indicating that HNC-3 has a high degree of reusability. These findings suggest that the addition of an acid may entirely restore the active sites of Pb^2+^ ions on HNC-3. This might be due to the rivalry between H^+^ and Pb^2+^ ions for adsorption sites [232]. Fan et al.‘s study [233] used 0.1 M Na_2_HPO_4_ and water to wash the nanoadsorbent to test the reusability of CMC-Fe_3_O_4_ for Pb^2+^ ion adsorption. The adsorption capacity slightly decreased as the number of adsorption and desorption cycles increased. The adsorption capacity of CMC-Fe_3_O_4_ was greater than 85% after five cycles. After five cycles, the saturation magnetization of CMC-Fe_3_O_4_ was 45.0 emu g^−1^, which is approximately equal to the previous saturation magnetization of 45.5 emu g^−1^ for the NPs. According to these findings, CMC-Fe_3_O_4_ is a very stable and reusable material, which makes it a desirable MNP for wastewater treatment. In an additional study, Martín et al. [234] assessed the feasibility of recovering Cu^2+^, Pb^2+^, and Zn^2+^ ions from ion-selective nanofiber materials across four cycles of adsorption and desorption. HNO_3_ (1.0 M) and H_3_PO_4_ (0.5 M) both gave good results, with over 90% ion recovery. No appreciable decrease in the adsorption capacity was observed for Pb ^2+^ and Zn^2+^ ions, suggesting that these nanofibers are readily recyclable and reusable. The adsorption capacity for Cu^2+^ ions, on the other hand, decreased over the adsorption–desorption cycles. Figure 16 shows a schematic diagram of the magnetic nanoadsorbent reuse procedure in aqueous solution.

### 2.4. Biological Materials as Adsorbents for Heavy Metal Removal

One new and emerging method for eliminating heavy metals from wastewater is biosorption. Even at low concentrations, this method is thought to be an effective detoxification approach for eliminating heavy metals. One type of adsorption process is biosorption, which involves both liquid and solid phases (solvent and sorbent). Both viable and nonviable organisms are needed to eliminate heavy metals. The primary benefit of using dead material rather than living material is that the former does not need growing conditions to flourish. The high effectiveness and low cost of this method make it suitable for the use of a variety of possible sorbents, including sawdust, seed shells, fungi, bacteria, yeast, and potato peels [235]. Biosorbents are believed to be useful, reasonably priced adsorbents that may be obtained as waste from a range of industry companies. Functional groups such as alcohol, ketone, carboxylic, aldehyde, and phenolic groups are known to increase the adsorption activity in the direction of metal removal. The metal ion concentration, adsorbent dose, temperature, pH, and contact duration are among the critical variables influencing the biomass potential in the adsorption process [236]. The primary biosorption mechanisms include surface precipitation, complexation, and ion exchange.

#### 2.4.1. Algal Biomass

Researchers have recently concentrated on using both live and non-living algal biomass to remove heavy metals from wastewater [237,238]. Living biomass has a limited adsorption capacity when it comes to removing heavy metals because this process occurs during the growth phase. This process is thought to be intracellular in nature and involves more complex adsorption mechanisms. Nevertheless, the extracellular process takes place in the biomass of non-living algae because the metals are adsorbed on the cell wall’s surface [238]. Environmental conditions such as pH, temperature, and contact duration, among others, might affect the ability of non-living algae to adsorb pollutants. The cell wall surface of algae has active functional groups that improve its biosorption capacity. El-Sheekh et al. investigated the use of marine macroalga *Pterocladia capillacea* in the eliminate of Cd^2+^ions. The results of using the adsorbent showed that the removal efficiency was 8.407mg g^−1^ with adsorption efficiency (96.047%). This study validates the possible application of *U. lactuca* green macroalga and its activated carbon for heavy metal removal from polluted water. This finding shows that *Pterocladia capillacea* have greater potential for Cd^2+^ion removal from aqueous solution [239]. Analogously, *Ulva flexuosa* biomass was used by Lekshmi R et al. [240] to evaluate its capacity for the biosorption of cadmium, cobalt, and zinc ions from wastewater environments. The removal efficiencies for zinc, cobalt, and cadmium were 90.8 ± 1.4%, 87.5 ± 2.3%, and 94.8 ± 3.3%, respectively. Brown seaweed *Sargassum carpophyllum* residue (SCR) and green seaweed *Caulerpa lentillifera* residue (CLR) were obtained after extraction of the bioactive polysaccharides by certain researchers, including Li et al., to remove Cu^2+^, Pb^2+^, Cd^2+^, and Mn^2+^ ions from water after modification with butanedioic anhydride. The maximum adsorption capacity for Mn^2+^, Cu^2+^, Cd^2+^, and Pb^2+^ and was 43.52, 52.37, 85.62, and 107.11 mg g^−1^, respectively. The maximal desorption achieved indicates the power of the adsorbent for metal ion removal [241]. Wastewater treatment and the production of biofuels are two environmental applications where algae have been discovered to be useful.

#### 2.4.2. Fungal Biomass

The large amount of cell wall components in fungal biomass adds to its potent sorbing ability. It can grow in unaltered surroundings. Chitin, glucan, mannan, proteins, and other polymers make up the cell wall, leading to increased adsorption capacity. Fungi can adsorb heavy metals through processes such as complexation, ion exchange, valence transformation, and intracellular precipitation [242]. Dried *Aspergillus niger* biomass was used by Cui et al. for biosorption of Pb^2+^, Hg^2+^, and Cd^2+^ ions. According to previous findings, pretreated dried biomass is a suitable absorbent for the removal of significant amounts of heavy metals, with high maximum capacities of 23.9, 27.2, and 21.5 mg g^−1^, respectively [243]. Recent work has focused on the removal of Pb^2+^, Hg^2+^, and Cd^2+^ ions by using *Aspergillus niger* spores, which are spherical particles with small diameters (2 µm) characterized by a negative charge. The biosorption of Pb^2+^, Hg^2+^, and Cd^2+^ from aqueous solutions using spores was analyzed at various biosorbent dosages, pH values, contact times, and initial heavy metal concentrations. The maximum biosorption capacities of Pb^2+^, Hg^2+^, and Cd^2+^ were 23.9, 27.2, and 21.5 mg g^−1^, respectively, at a natural pH, with initial concentrations of 30 mg/L. The order of the biosorption capacity for cationic heavy metals was Pb^2+^ > Cd^2+^ > Hg^2+^. The spores exhibited a short biosorption equilibrium time of 60 min at a pH range of 4.0–6.0, and the main biosorption mechanisms were electronic attraction, ion exchange, and complexation. In addition, spores can grow on many kinds of moist agricultural waste without any added nutrients. The results showed that spores could be considered potential biosorbents for the removal of cationic heavy metals from aqueous solutions.

Furthermore, Li Jiang et al. [244] used a novel marine *Pseudoalteromonas* sp. SCSE709-6 for the biosorption of Zn^2+^, Pb^2+^, and Cd^2+^ ions. *Pseudoalteromonas* sp. SCSE709-6 was successfully used as a competitive biosorbent for metals from aqueous solutions in a single system, as demonstrated by its highest adsorption capacities of 1.38, 1.05, and 0.91 mmol g^−1^ for Zn^2+^, Pb^2+^, and Cd^2+^ ions, respectively. The results revealed that carboxyl, amide, acyl, and hydroxyl groups were important for metal biosorption and that *Pseudoalteromonas* sp. SCSE709-6 performed better at removing metals from a variety of metal systems. Researchers have used a composite of fungal biomass and clay minerals, such as smectites, kaolinites, and bentonites, that indicate both chemical and physical stability when exposed to heavy metals.

According to Rashid et al. [245], Cr^6+^ ions may be removed from aqueous media via fungal biomass (WFB), which is from *Calocybe indica* fruiting bodies. It was concluded from the data that the adsorption process is more active and that it is an endothermic, spontaneous process. Researchers use fungi in two different forms: immobilized cells and mobilized cells. Owing to their ease of usage and handling, immobilized fungal cell systems have been utilized for metal absorption in the adsorption process in recent decades. The generated WFB adsorbent also has significant recycling potential. After five cycles of regeneration and adsorption. It can still maintain good remediation effectiveness of Cr^6+^ ions to 85.5%. For the purpose of removing metal ions from wastewater, Ding et al. [246] assessed the effectiveness of the adsorption process using immobilized *Aspergillus niger* cells. To prevent the loss of biosorbent during the regeneration phase, they immobilized the fungal biomass via polymer matrixes such as Ca–alginate gels. The results demonstrated that, in regard to the remediation of the previously stated heavy metals, the removal effectiveness of the immobilized biomass made of Ca–alginate was found to exhibit considerable potential for metal removal.

#### 2.4.3. Bacterial Adsorbent

Treating wastewater with bacteria is a good biological method. Owing to their lower size, greater availability, and adaptability, bacteria are a popular choice among researchers looking to eliminate heavy metals from wastewater. Ketones, aldehydes, and carboxyl groups are among the functional groups found in bacterial cell walls [247]. Typically, bacterial biomasses are employed to bind or support adsorbents in the process of removing heavy metals from aqueous solutions. To remove Cr^6+^ ions, Gupta et al. developed a batch system called simultaneous biosorption and bioaccumulation (SBB) that uses *Escherichia coli* biofilms immobilized on waste tea biomass (WTB) surfaces. At a starting concentration of 150 mg/L, both the SBB system and biosorption using WTB resulted in 99.99% removal of Cr^6+^ ions. However, for concentrations above 150 mg/L, the SBB system produced the maximum percentage removal, demonstrating that the SBB system is superior to biosorption via WTB at higher concentrations of toxic pollutants [248]. In another study, the process of removing metals was carried out via bacterial biomass. *Acinetobacter* sp. biomass was utilized to convert harmful As^3+^ ions. For live biomass, the adsorption maximum was 20.1 mg g^−1^. This outcome showed that there is a strong chance of removing ions [249]. In a different study, two strains, namely, *Nocardiopsis* sp. MORSY1948 and *Nocardia* sp. MORSY2014, which were isolated from the polluted region, were used. When the adsorbent dose was increased to 0.4%, these strains remediated the heavy metals. The greatest affinity was as follows: (90.37%) Zn^2+^ > (95.22%) Cr^6+^ > (93.53%) Ni^2+^ ions [250].

**Figure 17:**
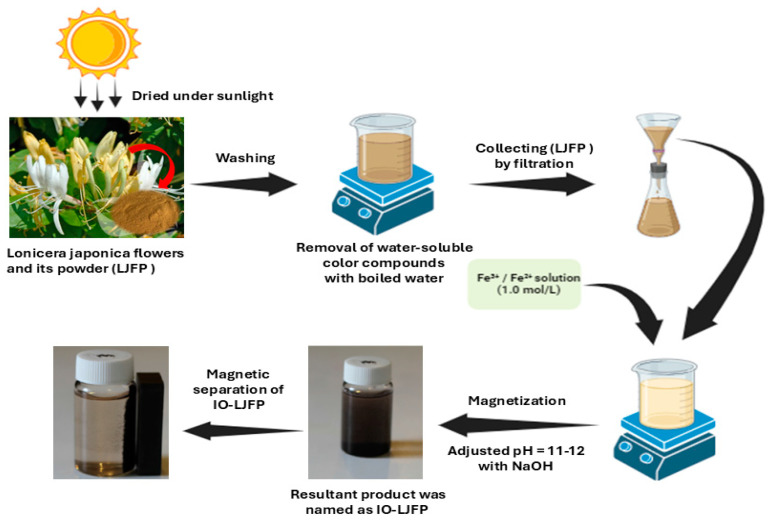
Schematic illustration of the preparation of iron oxide-impregnated LJFP (IO-LJFP) and the physical appearance of *Lonicera japonica* flowers (LJFs), their powder form (LJFP), and iron oxide-impregnated LJFP (IO-LJFP). Figure reproduced with permission of [253]. Copyright 2016, Elsevier.

Currently, scientists are concentrating on the use of plant roots for biosorption since they serve to identify the entire plant system and may be used in a phytoremediation technique called rhizofiltration [251]. For the biosorption of Cd^2+^ and Zn^2+^ ions, dry powdered roots of the wetland halophyte species *Kosteletzkya pentacarpos* growing in the presence or absence of 50 mM NaCl were used. In the biosorption procedure, the ideal parameters, such as a temperature of 25 °C and a contact period of 15 min, were determined to yield the highest adsorption. Cd^2+^ ions have greater sorption effectiveness (88.8%) than Zn^2+^ ions (56.9%) [252]. Heavy metals may be effectively removed from aqueous solutions via the use of magnetic porous biomass, an environmentally friendly detoxification material. For the purpose of removing Cu^2+^, Co^2+^, and Pb^2+^ ions from aqueous solutions, Lakshmi et al. synthesized a magnetized iron oxide-impregnated *Lonicera japonica* flower biomass (IO-LJFP), as shown in Figure 17. The use of IO-LJFP biomass is a promising, affordable, and effective method for removing heavy metal ions from industrial wastewaters, as shown by the overall results of this study [253]. Milojkovic et al. also used a compost derived from *Myriophyllum* spicatum to remove Cu^2+^, Cd^2+^, Ni^2+^, Pb^2+^, and Zn^2+^ ions in a selective manner. Because of its affordable, practical, and readily accessible qualities, they investigated whether this material may be utilized for heavy metal biosorption from wastewater [254]. In contrast to traditional adsorbents, heavy metal sequestration is facilitated by the binding groups present on the biomass of agricultural waste. The main benefit is in the application of agricultural biomass regeneration in an environmentally responsible manner. Table 5 contains a list of several types of literature on the utilization of plant material or agricultural biomass evaluated via the adsorption technique for the recovery of heavy metals from effluent.

## 3. Factors Influencing the Adsorption Process

Many variables, including the adsorbent dosage, starting concentration of metal ions, temperature, duration of contact, pH of the solution, and so on, may influence the adsorption of heavy metals. By optimizing these parameters, the adsorption of heavy metals may be significantly increased. Metal ion adsorption onto the adsorbent surface may be improved by keeping the parameters within the ideal range. We have discussed here the effects of the factors indicated above on the adsorptive removal of heavy metal ions.

### 3.1. Effect of the Adsorbent Dosage

Any substance that has the ability to absorb molecules from liquid, gaseous, or solid streams without changing is called an adsorbent. To obtain the maximum adsorption capacity under operating conditions, it is crucial to consider this parameter with respect to the adsorbent concentration [264]. Although the adsorption capacity decreases as a result of the aggregation of adsorption sites and a reduction in the surface area available for the adsorption of heavy metal ions, the adsorption percentage usually increases as the adsorbent dosage increases because more sites are accessible for adsorption [265]. Through adsorbent dosage optimization, it is possible to choose an adsorbent concentration that removes the most heavy metal ions with the least amount of adsorbent needed. However, the simultaneous use of many adsorbents decreases the adsorption rate because of the aggregation of particles and diminishes the overall surface area of the adsorbent [266].

**Figure 18:**
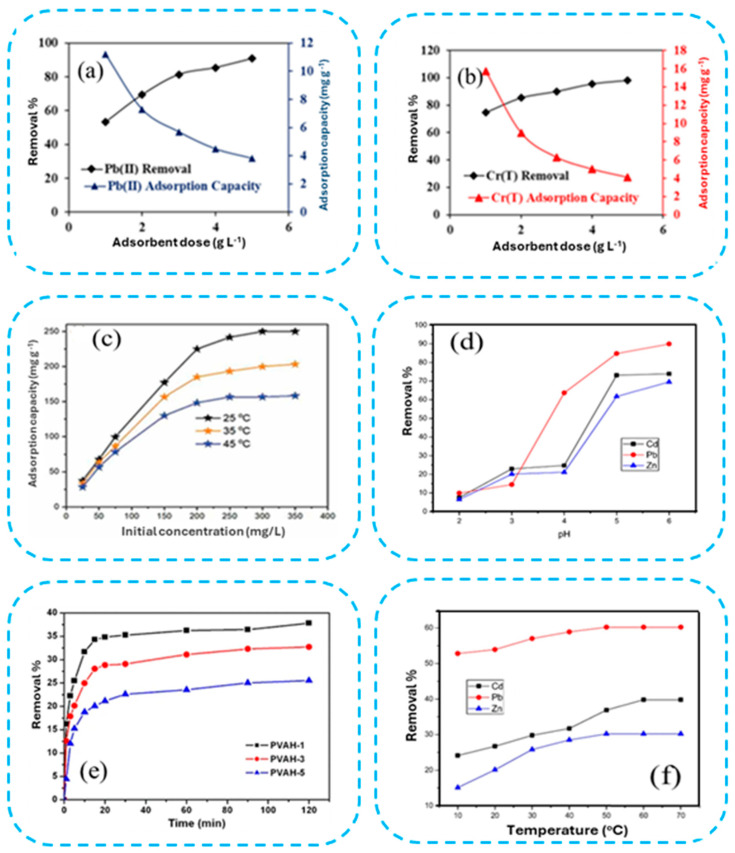
Various studies show the optimization of various parameters that affect (**a**,**b**) adsorbent dose, (**c**) initial ion concentration adsorbent dose, (**d**) pH, (**e**) contact time, and (**f**) temperature on the percentage removal of heavy metal ions by different types of nanomaterials. Figure reproduced with permission of [205,265,268,277,279]. Copyright 2016, 2020, and 2014, Elsevier, Springer.

Several studies on how the adsorbent dose influences the adsorption of heavy metal ions have been published in the literature. For example, in the adsorption of Cu^2+^ and Pb^2+^ ions, the ideal concentration of nanofibrous chitosan coated with TiO_2_ is 2000 mg/L. The partial deactivation of nanofiber surfaces is caused by NP aggregation at higher concentrations. Consequently, the adsorption capability of the adsorbent decreased significantly [267]. According to Lubna Jaber et al., when the Fe–GAC dosage was increased from 1 to 5 g/L, the adsorption efficiency increased from 53.3% to 90.8% for Pb^2+^ and from 74.8% to 98.2% for Cr^3+^ ions. This occurred because more Fe–GAC surface areas are available, and active adsorption sites are available with fixed concentrations of Pb^2+^ and Cr^3+^ ions in the water. However, the adsorption efficiency decreased as the Fe–GAC dose increased. This is most likely because the adsorption uptake and the Fe–GAC mass have an inverse relationship, as shown in Figure 18a,b [268]. Similarly, increasing the amount of polypyrrole (PPY) from 0.04 g to 0.08 g resulted in an increase in the adsorption percentage from 80% to 100%. This might be because there are more Cu^2+^ ions available per mass unit of PPY. However, the adsorption efficiency decreased to 60% when the PPY dose was increased to 0.12 and 0.16 g [269]. Peng et al. reported that when the Fe_3_O_4_ nanoparticle adsorption dosage was increased by 0.5–2 g/L, the adsorption efficacy increased from 41.7 to 92%. The enhanced discharge capacity is due to Pb^2+^ ions entering the dynamic regions of the Fe_3_O_4_ surface directly. The Fe_3_O_4_ dynamic surface area may be reduced by accumulation caused by high adsorbent fixation, which could explain the decreasing adsorption dosage with increasing adsorbent fraction [270]. Pb^2+^ ions have easier access to the active sites on the surface of the Fe_3_O_4_–carbon composite, which explains the increase in removal efficiency. The Fe_3_O_4_–carbon nanosorbent dose increased from 0.5 to 2 g/L, leading to a significant increase in the adsorption efficiency from 41.7 to 92%; however, this increase also caused a decrease in the adsorption capacity from 41.7 to 22.9 mg g^−1^. On the other hand, an increased adsorbent concentration could result in interparticle aggregation, which decreases the active surface area of Fe_3_O_4_@C and reduces its adsorption capacity when the adsorbent dose is increased [271].

### 3.2. Effects of the Initial Heavy Metal Ion Concentration

One of the most important factors in understanding good adsorption is the initial concentration of metal ions. When the concentration of metal ions increases, the rate of adsorption first increases; however, as the concentration increases further, the removal effectiveness decreases. At low concentrations, fewer metal ions are accessible for adsorption, resulting in a limited removal capacity because of the significant driving force for mass transfer at high starting concentrations of heavy metals. However, at higher concentrations, more ions are available for adsorption, leading to greater increases in the adsorption capacity and adsorption rate [272], which are dependent upon the potential interactions between the heavy metal ions and the adsorbent surface sites. However, after a certain initial concentration, more ions are available for the same number of adsorption sites, which decreases the removal effectiveness. Therefore, it is critical to optimize the concentration to ensure that the available adsorbent active sites and the initial metal ion concentration effectively bond. For example, at metal concentrations ranging from 50 to 700 mg/L, the scavenging of Fe^2+^, Mn^2+^, and Cu^2+^ ions onto SiO_2_/CuFe_2_O_4_/PANI was evaluated (Figure 18c). When the starting metal ion concentration was increased from 50 to 500 mg/L, the adsorption ability improved and eventually plateaued (i.e., the saturation state was reached) at 500 mg/L. The adsorption capacity of SiO_2_/CuFe_2_O_4_/PANI for Fe^2+^, Mn^2+^, and Cu^2+^ ions increased from 40.5 to 329.8 mg g^−1^, 35.9 to 307 mg g^−1^, and 27.5 to 179.9 mg g^−1^, respectively. The increased driving force from the pressure gradient was the reason for the increase in the adsorption capacity with increasing metal loading capacity. Saturation was reached when metal ions filled the available empty spaces in SiO_2_/CuFe_2_O_4_/PANI, achieving equilibrium [155]. In a different investigation, increasing the initial Hg^2+^ ion concentration on the surface of chitosan–alginate nanoparticles (CANPs) from 4 mg/L to 12 mg/L improved the adsorption rate, which consequently caused the removal of Hg^2+^ ions to decrease from 91% to 64% [273]. As the concentration of metal ions increases, Kalantari et al. reported that the adsorption efficacy decreases due to repulsive interactions between the adsorbed solute molecules in the bulk phase and the surface [274].

### 3.3. Effect of the Solution pH

By adjusting the pH, it is possible to ascertain the level of ionization and surface characteristics of an absorbent molecule. The initial pH of the solution affects the adsorption capacity and percent removal of metal ions when the other experimental parameters are held constant. The attraction and repulsion of the adsorbent to the metal ions can also be determined to modify the amount of adsorption. It also affects the ionization of adsorbent functional groups. The pH scale regulates the number of electrostatic charges that metal ions have, which impacts the rate of adsorption, making it a significant parameter [275]. A lower pH causes the adsorbent’s binding sites to protonate, which makes the adsorbent’s surface positively charged and reduces adsorption because positive metal ions and positive binding sites interact repulsively. An increased pH encourages positive metal ions to bond to the adsorbent’s negative surface, which increases adsorption [276]. An analysis of scientific data indicates that as the pH increases from 2.0 to 5.0, CANPs are better able to adsorb Hg^2+^ ions. This is because the electrostatic repulsion between the metal cations and the protonated functional moieties on the surface of CANPs inhibits the adsorption of Hg^2+^ ions at extremely low pH values. Additionally, the metal ions in water compete for adsorption with significant amounts of H^+^ and H_3_O^+^ ions. However, at moderate pH, the deprotonation of active sites reduces electrostatic repulsion, increasing the degree of adsorption [273]. Furthermore, at pH 4, the percentage of adsorption increased to 21.15%, 24.7%, and 63.7%, but at pH 2, the adsorption removal rates of Zn^2+^Cd^2+^ and Pb^2+^ ions were 6.5%, 7.5%, and 9.9%, respectively. Furthermore, increases of up to 69.5%, 73.9%, and 89.9%, respectively, were observed at pH 6 when the polythiophene–Al_2_O_3_ adsorbents were used, as shown in Figure 18d [277]. Pb^2+^ and Cu^2+^ ion adsorption on the chitosan/TiO_2_ nanofiber surface reached a maximum at pH 6.0 and decreased from pH 2.0 to pH 4.0 in a related study [267]. Similar research revealed that the percentage of Cd^2+^ ions adsorbed increased sharply between pH 4.0 and pH 6.0 and stabilized at pH 9.0 (97%). After increasing the pH to 11.0 once more, the adsorption percentage decreased to 80% [160]. Moreover, A. Modwi et al. produced a TiO_2_/CNNS composite that was used to remove Pb^2+^ ions from an aqueous solution. The results revealed that the adsorption efficacy reached a maximum at pH 3.0 and then steadily decreased as the pH increased. In conclusion, the Pb^2+^ ion adsorption capacity decreases from 75.2 to 62.5 mg g^−1^ when the pH increases from 3 to 8. At low pH values, Pb^2+^ ion is highly soluble and occurs as Pb^2+^ and Pb(OH)^+^. The metal hydroxide Pb(OH)_2_ is formed by Pb^2+^ and Cd^2+^ ions, and it precipitates at pH values higher than 6 and 8 [278].

### 3.4. Effect of the Contact Time

When choosing an adsorbent and calculating the binding speed, the contact time plays a crucial role in determining how long the equilibrium will take to remove all of the heavy metal ions from the solution. This factor is crucial for real-world applications because, as Figure 18e illustrates, the adsorption efficiency increases with time, reaches equilibrium at a certain optimal time, and then remains almost constant [279]. The effectiveness of Hg^2+^ ion removal on the CANP surface increased with increasing contact time from 0 to 90 min, as demonstrated by R. Dubey et al. In the first half hour, a rapid increase in Hg^2+^ ion removal efficiency was observed, which slowed after 90 min when the adsorption equilibrium was reached [273]. A related investigation revealed that within the first 20 min of contact, the Cd^2+^ and Pb^2+^ ions adsorbed 91% and 100% of the total amount of adsorption (48.53 and 53.33 mg g^−1^), respectively, on the surface of the MNCPs. After that, the process became independent of contact time since the rates of adsorption and desorption were in equilibrium [179]. In addition, the contact time of the adsorption process is influenced by chemical and physical characteristics. Qian et al. [280] reported that to reduce the contact time, an interparticle diffusion model is needed. Because of the functionalization of the chemical structure, PANI/PAV nanocomposites reportedly have a shorter contact duration than pure PANI [281].

### 3.5. The Effect of Temperature

The temperature of the solution is particularly crucial because it affects the spontaneity of reactions and temperature variations affect the removal of heavy metal ions [282]. Up to a certain limit, the adsorption effectiveness generally increases with temperature. The adsorption then either remains constant or starts to decrease, which could be caused by the heavy metal ions in the adsorbent starting to dissolve into the solution (Figure 18f) [277]. Depending on whether the adsorption process includes heat evolution or absorption, a subsequent temperature change can have one of two effects. In an exothermic process, the adsorption capacity decreases with increasing temperature, whereas in an endothermic process, it increases or remains constant [283]. Scientific research has shown that the adsorption of Hg^2+^ ions on chitosan–alginate nanoparticles (CANPs) is exothermic. The removal efficacy increased and reached its maximum value (approximately 79.40%) at 30 °C after the temperature increased from 10 °C to 30 °C. The decrease may occur further if the temperature increases and the metal ions become more mobile, which could cause the metal ions to desorb or decelerate from the adsorbent surface. Fewer metal ions were therefore adsorbed on CANPs as the temperature increased. The electrostatic interactions of the adsorbent with the Hg^2+^ ions also weaken with increasing temperature in the event of an exothermic adsorption process [273]. Further study demonstrated that as the temperature increased, the quantity of Pb^2+^ ions adsorbed increased. An endothermic adsorption mechanism is suggested by this increase. Pb^2+^ ion adsorption may increase with temperature because of increased ion mobility, which increases the number of ions interacting with active sites at adsorbent surfaces [284].

### 3.6. Effect of the Ionic Force

The solubility conditions of the ionic content play a crucial role in adsorption determination. It describes how the extra ions present in the liquid solution affect the ability of the adsorbed atoms to adsorb. When ions such as sodium and chloride ions are combined into the adsorbed arrangement, they are concentrated. The strength of the ions determines how much influence they have because of the competition for active sites on the adsorbent surface between the target ions and the extra ions, as well as the coulombic potential screening between the adsorbent molecule and the adsorbing ions. The adsorption efficacy is dependent on both the affinity of the adsorbent and the concentration of extra ions. The adsorption efficiency of the waste material is unaffected by the ionic strength of the solution when the adsorbent’s affinity for target metal ions is greater than that of added ions. The ionic strength has little effect on the adsorption of heavy metal ions, according to a few studies [285]. Hasanzadeh et al. demonstrated how NaCl at a concentration of 3 mol/L affected the adsorption of two heavy metal ions on the surface of MNCPs: Pb^2+^ and Cd^2+^ ions [179]. Compared with Pb^2+^ and Cd^2+^ ions, the adsorbent is less effective when sodium ions are applied. NaCl did not affect the enhanced sorption of large metallic particles. NaCl’s ability to improve the separation of advantageous aggregations outside the adsorbent increases the adsorption limit, which helps to further support this theory. Therefore, a decrease in ionic activity was predicted to have an effect on adsorption. The impact of ionic quality thus likely changes depending on the NaCl concentration and the features of the adsorbed particle’s adsorbing surface. Another study by Xu et al. [154] revealed that adding NaCl to Fe_3_O_4_-SiO_2_-GSH MNPs slightly increased the adsorption capacity of Pb^2+^ ions at a concentration of 0.025 mM; however, adding more NaCl up to 0.2 mM caused the adsorption capacity to decrease from 98.87 to 85.72 mg g^−1^. The rationale for the study’s findings is that NaCl increases the adsorption capacity by first encouraging the dissociation of functional groups on the adsorbent surface. The competition between ions for binding sites was the cause of the latter decrease. Thus, the effect of ionic strength might be small to large, depending on the NaCl concentration and the affinity of the adsorbent surface for adsorbate molecules.

## 4. Kinetic Models of Metal Ion Surface Adsorption on Various Nanomaterials

While equilibrium studies offer valuable insights into the maximum adsorption capacity of materials, they fall short in addressing the dynamic aspects of adsorption processes, which are crucial for industrial applications. Even the most effective sorbents may be unsuitable for large-scale use if the adsorption process occurs too slowly. This is where kinetic studies play a pivotal role, as they provide a deeper understanding of the rate at which adsorption occurs, revealing the time-dependent behavior of the process. Such insights are essential for designing efficient industrial systems, where time constraints are often critical.

Unfortunately, in many adsorption studies, the kinetic aspect is treated superficially, typically involving the measurement of a single kinetic curve and fitting it to one of the common models described below. While this approach is not incorrect, it often lacks the depth required to fully understand the adsorption dynamics. A single kinetic equation is rarely sufficient for designing industrial-scale installations, as it does not account for the influence of key factors such as temperature and concentration, both of which are fundamental in adsorption equilibrium studies. Despite this, temperature-dependent adsorption rates are rarely examined in the literature, leaving a significant gap in the understanding of how external factors influence adsorption kinetics.

To address this gap, the following section introduces and explains the most commonly applied kinetic models in the context of metal ion surface adsorption on various nanomaterials. These models not only help us comprehend the mechanisms underlying adsorption but also guide the optimization of industrial processes by predicting adsorption rates under different conditions. 

When referring to kinetic models of metal ion surface adsorption on various nanomaterials, we mean the mathematical frameworks that describe how metal ions interact with and adhere to nanomaterial surfaces over time. Several kinetic models are widely employed to elucidate both the mechanisms and rates at which adsorption occurs, which is crucial for applications such as water purification, catalysis, and sensing. The following models are the most commonly used to analyze the adsorption rate in various systems.

1. Pseudo-First-Order Model (PFO): This model assumes that the rate of adsorption is directly proportional to the number of vacant sites on the adsorbent surface. This model is often applied to systems where the adsorption rate is influenced by the concentration of the adsorbate. Equation (1) shows the linear form of the integrated rate equation for the PFO model [286].
  (1) $$\log\left(q_{e}-q_{t}\right)=\log q_{e}-\frac{k_{1}}{2.303}t$$
2. Pseudo-Second-Order Model (PSO): This model posits that the rate of adsorption is proportional to the square of the number of vacant sites, in contrast to the first order. This model assumes that the adsorption process may be chemisorption involving valence forces through sharing or exchange of electrons. It often provides a better fit for systems where the adsorption capacity is more dependent on the availability of adsorption sites. The equation for this model is as follows:
  (2) $$\frac{t}{q_{t}\;}=\frac{1}{k_{2}{q_{e}}^{2}}+\frac{t}{q_{e}}$$
  where q_t_ and q_e_ are the adsorption capacity at time t and at equilibrium (mg g^−1^), respectively. Additionally, k_1_ and k_2_ are the rate constants of PFO adsorption and PSO, respectively. One can determine k_1_ and q_e_, respectively, from the slope and intercept of the plot of log (q_e_ − q_t_) vs. t. The values of k_2_ and equilibrium adsorption capacity (q_e_) for pseudo-second order may be determined from the slope and intercept, respectively, of the linear plot of t/q_t_ vs. t.
3. Intraparticle Diffusion Model: This model considers that adsorption occurs in multiple steps, including diffusion through the boundary layer and into the pores of the adsorbent [287]. It is often seen in mesoporous materials like activated carbon or modified silica nanoparticles. For instance, with activated carbon modified for enhanced metal ion adsorption, the intraparticle diffusion model can be particularly informative, as diffusion within the pore structure can control the adsorption rate. By applying this model, we can distinguish between surface-bound and pore-diffusion-controlled adsorption phases in these materials. The equation can be written as follows:
  (3) $$qt={\;K}_{p}t^{0.5}+C$$
  where K_p_ represents the intraparticle diffusion rate constant (mg/g min^0.5^), C is the boundary thickness, and t^0.5^ is the half-life time in seconds. To recognize that the intraparticle diffusion has been obeyed, the plot should be linear, and only if the lines pass through the point of origin is intraparticle diffusion considered the rate-controlling step. But when the plot fails to pass through the origin, the implication is that intraparticle diffusion may not be the only rate-limiting step. Other kinetic models may be responsible for controlling the adsorption rate due to their input to the net transport of the adsorbate ions.
4. Elovich Model: This model has been reported as compatible with describing the adsorption system when the adsorbate species and the adsorbent sites interact chemically via a second-order mechanism [288]. Representation of the linear equation of the Elovich kinetic model is as follows:
  (4) $$qt=\frac{1}{\beta }\ln(\beta \alpha )+\frac{1}{\beta }\ln\left(t\right)$$
  where q_t_ (mg g^−1^) is the adsorbed adsorbate amount at time t, α is the initial rate constant (mg g^−1^ min^−1^), and β is the desorption constant (g/mg).

The choice of kinetic model reflects not only the nanomaterial’s physical structure but also its functionalization. For instance, functionalized nanoparticles with active surface groups often follow the PSO model, while porous materials may better align with the intraparticle diffusion model due to the diffusion process within their pore networks. Studies show varied kinetic behaviors of metal ion adsorption on nanomaterial surfaces. For example, Table 6 provides a comparative summary of recent applications of these models, illustrating their effectiveness for different nanomaterial types under varying conditions.

Table 6 highlights several studies where various kinetic models were applied to describe the adsorption of metal ions on nanomaterials. The pseudo-second order (PSO) model was the most commonly fitted model across these studies, reflecting its widespread applicability in describing chemisorption processes. However, it is worth noting that some studies listed in Table 6 applied both the PSO and the intraparticle diffusion models simultaneously. This dual-model approach raises concerns, as a single adsorption phenomenon cannot be accurately described by two different models. The apparent agreement between these models is likely due to a lack of comprehensive kinetic investigations, which are crucial to identifying the rate-limiting step in the adsorption process. In extreme cases, it is possible that the authors are investigating systems operating in a mixed control regime, where both diffusion and the adsorption process occur at comparable rates. In such scenarios, the system could shift to either full kinetic control or full diffusion control with changes in operational conditions, such as the mixing dynamics. Therefore, the proper construction of a mathematical model must take into account not only adsorption kinetics but also the fluid dynamics involved in the system. 

It is precisely the complexity of phenomena at the liquid–solid interface that makes adsorption studies uniquely challenging. Without a deeper understanding of the dynamic interplay between diffusion, adsorption, and fluid movement, it is difficult to develop a comprehensive model that accurately reflects real-world industrial processes. This is why advancing kinetic research is so essential, as it allows for more precise identification of controlling factors and, consequently, better design of industrial adsorption systems.

## 5. Adsorption Isotherms

Adsorption isotherms usually provide information about the connection between the adsorbent and the adsorbate at a constant temperature under equilibrium parameters. Adsorption isotherms are another tool used to calculate the maximum adsorption capacity. Using Langmuir, Freundlich, Temkin, and Dubinin–Radushkevich adsorption isotherms, the adsorption of metal ions onto nanocomposites was studied [289].

### 5.1. Langmuir Isotherm Model 

The homogenous single-layer adsorption on a surface is described by this isotherm. It is assumed that the adsorption process takes place at similar and energetically equivalent active sites, even at adjacent sites, free from steric hindrance and side interactions between the adsorbed molecules. Every molecule has a constant activation energy for adsorption and enthalpy [290,291].

The Langmuir equation is as follows:

(5) $$\frac{C_{e}}{q_{e}}=\frac{1}{K_{L}{\;q}_{m}}+\frac{C_{e}}{q_{m}}$$

where q_e_ (mg g^−1^) is the amount of metal ions adsorbed at equilibrium, and C_e_ (mg/L) is the equilibrium concentration of metal ions in solution. The maximum adsorbent capacity for monolayers is expressed as q_m_ (mg g^−1^) and the Langmuir constant of adsorption is denoted by K_L_. The linear plot of C_e_/q_e_ vs. C_e_ gives the values with a slope and an intercept. 

### 5.2. Freundlich Isotherm Model

The Freundlich isotherm model is used to characterize reversible and nonideal adsorption processes. The Langmuir isotherm presumes that adsorption occurs in a monolayer, whereas the Freundlich isotherm can be used for heterogeneous systems, permitting adsorption on many layers. Owing to the heterogeneity of the surface, this model acknowledges that the adsorption heat may vary rather than assume a constant adsorption heat across it.

Furthermore, the Freundlich isotherm accounts for the varying binding affinities within the adsorbent. As the adsorption process progresses, the more energetically favorable (strongest) binding sites are occupied first, leading to a decrease in the overall adsorption energy as the remaining weaker sites are filled. This behavior reflects the nonideal and complex nature of many real-world adsorption scenarios [292]. The model is succinctly expressed by the following Equation (6):

(6) $$\ln q_{e}=\ln K_{F}+\frac{1}{n}\ln C_{e}$$

The two terms K_F_ adsorption capacity (Freundlich constant) and n adsorption intensity can be found from the intercept and slope, respectively, of the plot of ln q_e_ vs. ln C_e_. The system of adsorbent and adsorbate is favorably characterized by the value of n. When 0 < 1/n < 1, the adsorption is advantageous; when 1/n = 0, it is irreversible; and when 1/n > 1, it is unfavorable [293].

### 5.3. Temkin Isotherm Model

The Temkin isotherm model, which can be represented by the linearized form of Equation (7), was used to assess the degree of adsorption heat and bonding energies of the molecules in the layer during sorbate and sorbent interactions [294]:

(7) $$q_{e}=\frac{\mathrm{RT}}{b_{T}}\ln K_{T\;}+\frac{\mathrm{RT}}{b_{T}}\ln C_{e}=B_{T}\ln\left(K_{T}C_{e}\right)\;$$

where constant B_T =_ $\frac{\mathrm{RT}}{b_{T}}\;$, which is related to the adsorption heat, R is the gas constant (8.314 J/mol K), and T(K) is the absolute temperature in Kelvin. The change in adsorption energy is represented by the Temkin isotherm constant b_T_ (J/mol), and the equilibrium binding constant (K_T_) corresponds to the maximum binding energy. Based on q_e_ against ln C_e_, the slope and intercept of the linear plot may be used to compute B_T_ and K_T_, respectively.

### 5.4. Dubinin–Radushkevich (D-R) Isotherm Model

The D-R isotherm addresses the adsorption of vapors on solids. Polanyi’s theory was used to represent the adsorption mechanism onto heterogeneous surfaces as well as the Gaussian energy distribution. The D–R isotherm suggests that adsorption does not take place on the pore walls layer by layer but that the micropores are filled instead. Compared with the Langmuir and Freundlich isotherms, the D-R model performs better. Durbin’s equations are typically used to distinguish between chemical and physical adsorption since they do not require a homogenous surface or a constant adsorption potential. The matching formulas are as follows:

(8) $$\varepsilon \;=\;\mathrm{RT}\;\ln\;\left(1+\frac{1}{C_{e}}\right)$$

(9) $${\ln\;q}_{e}=\ln q_{s}-{\;\beta \varepsilon }^{2}$$

where the adsorption potential is represented by ε (kJ mol^−1^). The quantity of metal ions adsorbed at equilibrium is given by q_e_ (mg g^−1^). The maximum quantity of metal ions that can be adsorbed on the nanocomposite is denoted by q_s_ (mg g^−1^). Equation (10) relates the constant β to the adsorption energy (E). The values of q_s_ and β were computed by graphing ln q_e_ against ε^2^. The energy of adsorption is presented as follows:

(10) $$E=\frac{1}{{\left(2\beta \right)}^{0.5}}$$

The physical or chemical nature of adsorption is indicated by the value of E. When E is less than 8 kJ mol^−1^, physical adsorption occurs. When 8 < E < 16 kJ mol^−1^, chemical adsorption is indicated [295]. Numerous investigations focused on the adsorption of metal ions onto the surfaces of various nanomaterials have been reported in scientific publications. Table 7 contains an overview of certain studies.

## 6. Conclusions and Outlook

Currently, there are greater risks to public health and the environment than ever before, making the removal of heavy metals from wastewater imperative. Numerous studies have been conducted to increase drinking water quality via a variety of techniques, including adsorption, chemical precipitation, coagulation/flocculation membrane filtration, electrochemical treatment, flotation, and ion exchange for the removal of heavy metals, to comply with the proposed environmental regulations. Adsorption is a simple and efficient physicochemical technique that eliminates heavy metals from wastewater. Thus, creating adsorbents that offer easy recovery, high adsorption capacity, strong selectivity, safety, and cost-effectiveness has long been a focal point of research. Recent developments in nanoscience and nanotechnology have opened the way to various effective, affordable, and environmentally benign methods for removing environmental pollutants. Numerous nanomaterials have physicochemical characteristics that make them viable options for purifying wastewater. NPs have the ability to eliminate metal ions even at low concentrations because of their high selectivity and adsorption capacity. 

To combat water pollution, this work provides a broad overview of the various nanostructured materials used as adsorbents to eliminate heavy metal ions, such as metals, metal oxides, magnetic nanocomposites, and carbon nanomaterials. The adsorption process is influenced by many factors, such as temperature, ionic strength, surface area, porosity of the material, affinitive functional group sites, pH, and adsorbent dose. 

The development of effective adsorption nanomaterials for the removal of heavy metal ions from wastewater has advanced significantly, yet several challenges remain. One key research gap is the limited selectivity of adsorbents in complex multi-component systems, where multiple metal ions compete for adsorption sites, which limits the efficiency of current adsorbents. Furthermore, the reusability and regeneration efficiency of many adsorbents decline after multiple use cycles, which hampers their practical application on an industrial scale. Another critical gap is the aggregation of nanomaterials during adsorption, which reduces their surface area and effectiveness.

Looking ahead, magnetic composites offer a high surface area and can be separated easily from aqueous solutions by applying an external magnetic field. This is the best choice to minimize the aggregation of nanoparticles, difficult separation, and reusability issues compared to traditional adsorbents. So, promising research directions should focus on the functionalization of nanomaterials, such as magnetic nanocomposites, to enhance their selectivity for hazardous ions and improve their regeneration capabilities. Surface functionalization via a variety of compounds, including inorganic, organic, polymeric, and protein can increase the affinity of adsorbents for target ions, while maintaining their adsorption capacity even in the presence of competing ions. Additionally, the development of scalable synthesis methods that are both cost-effective and environmentally friendly is crucial for the widespread adoption of these materials. The use of green chemistry approaches and sustainable material design can further reduce the environmental footprint of adsorbent production and can be applied on a large scale, from the laboratory to the ground, to adsorb different kinds of environmental pollutants, which would improve pollution control on a large scale for commercial applications.

This review ends with an encapsulating forecast of the considerable potential of these nanomaterials for future use in wastewater treatment to remove heavy metal ions. This article provides future workers with comprehensive knowledge about the investigated topic area, providing detailed and comparative data about the suitability of different kinds of nanoparticles for removing heavy metal ions from wastewater. Therefore, this article not only sheds light on a variety of topics already investigated by scientists but also assists them in creating their own research challenges by considering the applicability and consequences of their findings for both industry and science.

## Figures and Tables

**Table 1:** Common sources and impacts of heavy metals on human health.

Heavy Metal	Human Health Impacts	Permissible Limit (WHO) (mg/L)	Common Sources	Reference
Arsenic (As)	Carcinogenicity, circulatory system difficulties, muscular weakness, nerve irritation, protein coagulation, skin injury	0.01	Agriculture applications, coal-fired and geothermal power generation, electronic manufacture, light filters, metallurgy, naturally occurring, nonferrous smelters, pyrotechnics, tanning, pigments, and veterinary medication	[14]
Cadmium (Cd)	Carcinogenicity, DNA damage, gastric discomfort, hyperactivity, kidney damage, and renal failure	0.003	Alloys, anticorrosive metal coatings, coal combustion, naturally existing, different chemical areas, phosphatic fertilizers in agriculture, pigments, and plastic stabilizers	[15]
Chromium (Cr)	Allergic dermatitis, damage to the kidneys and liver, diarrhea, headaches, nausea, neurotoxicity, and vomiting	0.1	Chemical-based industries, environmentally occurring, metallurgy of steel production, passivation of cooling circuit corrosion, pigments, plating, refractory, tanning of textiles and leather, and wood treatment	[16]
Copper (Cu)	Anorexia, liver and kidney damage, digestive problems, and central nervous system irritation; can harm a number of aquatic animals and cause sadness	1.3	Alloys, copper mining, naturally existing materials, pigments, smelting, steel production, steel processing, machinery manufacturing, and tools for chemistry and pharmacy	[17]
Silver (Ag)	Corrosion and damage of the lung, sore throat, skin damage, and mucosal irritation	0.1	Industrial scrap and mining	[18]
Lead (Pb)	Carcinogenicity, diminished brain development, excessive blood pressure, and kidney damage	0.05	Alloys, anti-knock materials, batteries made of lead, cable sheathings, home plumbing systems, ceramics, glass, paints, plastic, sheets, and solder	[19]
Mercury (Hg)	Abortions, carcinogenicity, gastrointestinal problems, gingivitis, kidney damage, nerve system damage, and stomatitis; acute effects on the liver, brain, lung; and hair loss and memory loss	0.001–0.005	Catalysts, combustion of fossil fuels, dental fillings, electronic industries, electrical and measuring equipment, fluorescent light bulbs, pharmaceuticals, rectifiers, scientific instruments, solders, and oscillators	[20,21]
Nickel (Ni)	Carcinogenicity, coughing, hair loss, nausea, and persistent asthma	0.015	Arc welding, batteries, catalysts, computer parts, dental and surgical prostheses, electroplating, fertilizer plating, molds for glass and ceramic containers, paper products, paint, and ceramic pigment rods	[22]
Zinc (Zn)	Depression, hyperactivity, increased thirst, neurological symptoms, and symptoms such as physical dysfunction and tiredness	5.0	Agricultural uses (phosphatic fertilizers), anticorrosion coatings, batteries, cans, coal, mining, paints, PVC stabilizers, rubber industries, steel processing, soldering, waste combustion, and welding fluxes	[23]
Uranium U(VI)	Uranium deposition areas in the body are the kidneys, the liver, and the bones; the toxicity of uranium is a function of the route of exposure, particle solubility, contact time, and route of elimination	0.03	Anthropogenic sources include phosphate fertilizers, mine waste, fly ash from power plants, and military use; uranium occurs in oxidation states III, IV, V, and VI but the dominant forms in nature are IV and VI.	[24]

**Table 2:** Different methods for synthesizing iron oxide nanoparticles under various conditions.

Method	Iron Oxide Nanoparticle	Size (nm)	pH	Temperature (°C)	Reference
Coprecipitation	Fe_3_O_4_	7.8	11	45	[125]
Sonochemical coprecipitation (ultrasound assistance)	Fe_3_O_4_	13			[126]
Coprecipitation	Fe_3_O_4_	10–20	9	80	[127]
Ultrasonic-assisted chemical coprecipitation	Fe_3_O_4_	15	7	60	[128]
Sol–gel	Fe_3_O_4_	2.02	--	200	[129]
		5.58		400	
		8.35		600	
Hydrothermal	α-γ Fe_2_O_3_	310	--	200	[130]
Solvothermal	γ-Fe_2_O_3_	2	--	130	[131]
		4.5		180	
		6.1		200	
Solvothermal	γ-Fe_2_O_3_	3000	--	400	[132]
Mechanochemical milling	α-Fe_2_O_3_	17.1	--		[133]
Ultrasonic spray pyrolysis	α-Fe_2_O_3_	18	--	400	[134]

**Table 3:** The impact of surface area on the removal efficiency of metal ions by magnetic adsorbents.

Magnetic Adsorbent	Metal Ion	Surface Area (m^2^ g^−1^)	Particle Size (nm)	Removal Efficiency %	References
CaO/Fe_3_O_4_/SDS	Cr^3+^	36.47	89	98.7	[141]
Cobalt ferrite	Cr^3+^	17.84	500	100	[142]
	Cd^2+^			100	
Bentonite/Fe_3_O_4_	Cd^2+^	105.44	64.5	96.56	[143]
	Pb^2+^			98.32	
	Ni^2+^			94.66	
Fe_3_O_4_/talac	Ni^2+^	37.079	—	50.23	[144]
	Cu^2+^			72.15	
Maghemite NPs	Cu^2+^	175	100	96.5	[145]
Fe_3_O_4_@Zn	Hg^2+^	15	100	94.5	[146]
Magnetic chitosan nanoparticles	Hg^2+^	—	20-30	99.8	[147]
CaO/Fe_3_O_4_	Pb^2+^	71.23	219	97.24	[148]

**Table 4:** Conditions of the experiment for removing heavy metal ions with various nanoadsorbents.

Adsorbents	Metal Ions	Temperature (°C)	pH	Initial Ion Conc. (mg L^−1^)	Contact Time (min)	Adsorption Capacity (mg g^−1^)	References
Al_2_O_3_	Cu^2+^	25	5	20	60	-	[183]
Polyaniline/Al_2_O_3_	Pb^2+^ Cr^6+^	25	6 2	-	-	9.90 12.04	[184]
Al_2_O_3_/MWCNTs	Cd^2+^	25	7	1000	120	27.21	[185]
MnO_2_-coated carbon nanotubes (MnO_2_/CNTs)	Hg^2+^	50	5–7	10	80	58.80	[186]
Polythiophene/MnO_2_ composite	Pb^2+^ Zn^2+^ Cu^2+^	25	-	400	180	82.10 30.72 60.00	[187]
PAN/PPy/MnO_2_) nanofiber	Pb^2+^	25	6	400	120	274.73	[188]
TiO_2_/PANI composite	Pb^2+^	25	7	200	180	1499.99	[189]
PANI/TiO_2_ composites	Cr^6+^	25	7–8	600	1.5–1024	394.43	[190]
Chitosan–SiO_2_–TiO_2_ nanocomposite	Cr^6+^	25	3	100	120	182.43	[191]
Superabsorbent polymer hydrogel (SPH)	Cd^2+^	25	7	100	–	1979.6	[192]
Modified and raw MWCNTs	Cr^6+^	25	3	1	1400	1.31 3.11	[193]
CNTs	Pb^2+^ Cu^2+^ Cd^2+^	25	5	30	–	97.08 24.49 10.86	[194]
GO-DPA	Pb^2+^ Cd^2+^ Ni^2+^ Cu^2+^	25	5	20	240	369.7 257.2 180.8 358.8	[195]
EDTA-GO	Pb^2+^	25	6.8	100	120	479.0	[196]
GO–aminosiloxane	Pb^2+^	30	4-5	100	120	312.0	[197]
Fe_3_O_4_ NPs@AC composite	Pb^2+^ As^3+^ Cd^2+^	25	5.5	20	10	147.05 151.51 119.04	[176]
Ca-substituted nickel zinc nanoferrites	Cd^2+^ Cr^2+^	25	6	0.989 1.28	10	128.20 23.54	[120]
(ZFN-Alg beads)	Pb^2+^ Cu^2+^	35	6	50	90	108.8 106.6	[198]
Spinel nanoparticles (NPs)	Cd^2+^ Pb^2+^	25	2 12	25	720	157.7 63.10	[199]
Magnetic ferrite nanoparticles	Cu^2+^	25	8	25	180	124.80	[200]
Ultrafine mesoporous magnetite (Fe_3_O_4_) nanoparticles (UFMNPs)	Pb^2+^ Cd^2+^ Cu^2+^ Ni^2+^	25	5.5	10	180	85.00 79.00 83.00 66.00	[201]
Magnetic MNP@SiO_2_ nanocomposites	Pb^2+^	25	4–8	10	180	14.90	[202]
Fe_3_O_4_/MnO_2_ composites	Cu^2+^ Cd^2+^ Pb^2+^ Zn^2+^	30 30 30 60	6.5	10	1440	498.0 439.0 416.5 490.0	[203]
Carbon magnetic nanocomposites	Cu^2+^ Pb^2+^ Zn^2+^	25	5.8	10	240	81.36 83.54 57.11	[204]
Fe_3_O_4_@AMPA nanocomposite	Cd^2+^	25	7	250	150	265.0	[205]
Fe_3_O_4_@PAA@TSH MNPs	Pb^2+^ Cd^2+^	25	5.2	100	40	188.7 107.5	[206]
Fe_3_O_4_@carbon nanocomposite	Pb^2+^ Cu^2+^	27	6.5	30	120	151.5 48.08	[207]
Magnetic chitosan/Al_2_O_3_/Fe_3_O_4_	Cd^2+^ Cu^2+^ Zn^2+^	25	5.3	10	300	85.79 35.97 32.89	[208]
Fe_3_O_4_@SiO_2_-NH-pyd	Pb^2+^	25	7	10	20	72.0	[209]
Amino-functionalized magnetic graphene composite	Pb^2+^ Hg^2+^ Cd^2+^ Ni^2+^	20	6–7	5	120	27.95 23.03 27.83 22.07	[210]
Magnetic nanocomposite (HMNC)	Cr^6+^	40	2	150	120	301.2	[211]
MoS_2_/Fe_3_O_4_ nanocomposite	Hg^2+^	25	6-7	100	180	1923.5	[212]
CoFe_2_O_4_@SiO_2_–SDS–DTO magnetic nanoparticles	Cd^2+^	25	6	200	30	350.08	[213]
Bentonite/Fe_3_O_4_	Cd^2+^ Pb^2+^ Ni^2+^	25	6	5	20 20 30	108.68 9.43.0 5.98	[214]
Fe_3_O_4_–chitosan@bentonite	Cr^6+^	25	2	60	120	62.10	[215]
GO/Fe_3_O_4_	Pb^2+^	50	6.2	5	-	126.6	[216]
TiO_2_/SiO_2_/Fe_3_O_4_	Cd^2+^ Hg^2+^ Ni^2+^	25	2	20	40	670.9 745.6 563.0	[217]
Glycol–Fe_3_O_4_	Pb^2+^	25	6	100	10	-	[218]
Fe_3_O_4_@SiO_2_-NHMFL	Pb^2+^ Cu^2+^	25	5	50	0.5	150.33 70.7	[219]
APTES-Fe_3_O_4_ (3wt%)	As^5+^	25	2	1	210	14.60	[220]
CoFe_2_O_4_@SiO_2_-EDTA	Hg^2+^	25	7	20	360	103.3	[221]
CMC/SA/graphene oxide@ Fe_3_O_4_	Cu^2+^ Cd^2+^ Pb^2+^	30	5	100	1400	55.96 86.28 189.04	[222]
Proanthocyanidin-functionalized Fe_3_O_4_	Cu^2+^ Cd^2+^ Pb^2+^	25	8	20	30	18.8 20.9 21.5	[223]
CuFe_2_O_4_/polyaniline composite	Hg^2+^	25	7	0.025	240	157.1	[224]
MWCNT/γ-Fe_2_O_3_ MWCNT-PEI/γ-Fe_2_O_3_	Cr^6+^	30	4	500	150	208.1 352.3	[225]
(Fe_3_O_4_-BA and Fe_3_O_4_-FP) nanocomposite	Pb^2+^	25	7	15	3-15	27.85	[226]
Polyaniline/Fe_3_O_4_	Pb^2+^	25	9	50	120	111.11	[227]
Iron oxide composite	As^2+^ Pb^2+^ Cd^2+^	25	5-6	10	90-120	144.70 128.01 122.10	[228]
Fe_3_O_4_–chitosan composite	Pb^2+^	50	7	0.3	60	50.72	[229]

**Table 5:** List of plant material (agricultural biomass) employed in heavy metal removal.

Adsorbent	Metal Ion	pH	Time (min)	Adsorption Capacity (mg g^−1^)	References
Banana peel	Cu^2+^ Pb^2+^ Cd^2+^ Cr^2+^	4	30	49.5 45.5 30.7 25.2	[255]
Oak acorn peel	Cr^6+^	7	420	47.39	[256]
Picea smithiana sawdust	Pb^2+^ Cr^6+^ Cd^2+^	8	60	6.35 3.37 2.87	[257]
Modified sawdust	Cr^6+^	2	-	8.84	[258]
Cashew nut shell	Cu^2+^	5	30	20.0	[259]
Peanut shells	Pb^2+^ Cu^2+^ Cd^2+^ Ni^2+^	4	60	239.2 147.8 188.6 119.96	[260]
Sunflower leaves	Cd^2+^	2-7	5-40	7.60	[261]
Orange peel	Co^2+^	5.4	100	4.25	[262]
Litchi chinensis seeds	Ni^2+^	7.5	264	66.62	[263]

**Table 6:** Different kinetic models for metal ion adsorption by various types of nanomaterials.

Nanomaterials	Metal Ion	Kinetic Models	References
Chitosan/TiO_2_ nanofibers	Cu^2+^ Pb^2+^	PFO	[267]
Fe_3_O_4_/MnO_2_ composites	Pb^2+^ Cu^2+^ Cd^2+^ Zn^2+^	PSO	[203]
Carbon magnetic nanocomposites	Cu^2+^ Pb^2+^ Zn^2+^	PSO	[204]
CuO NPs	Cr^6+^	PSO	[59]
Fe_3_O_4_ NPs	Cu^2+^	PSO and intraparticle diffusion	[167]
Glycine-functionalized magnetic nanoparticles (GFMNPS)	Pb^2+^	PSO and intraparticle diffusion	[61]
Fe_3_O_4_@SiO_2_-NH-pyd	Cu^2+^ Pb^2+^	Diffusion model	[209]
Al_2_O_3_/MWCNTs	Cd^2+^	Diffusion model	[185]

**Table 7:** Models of adsorption isotherms for metal ion adsorption by different types of nanomaterials.

Nanomaterials	Metal Ion	Adsorption Isotherm	References
Amino-functionalized magnetic nanoparticles	Cu^2+^ Cr^6+^	Langmuir Langmuir	[182]
MNP-DMSA nanoadsorbents	Pb^2+^ Ni^2+^ Cd^2+^	Langmuir	[165]
Al_2_O_3_/MWCNTs	Cd^2+^	Langmuir	[185]
CuO NPs	Cr^6+^	Freundlich	[59]
Fe_3_O_4_/MnO_2_ composites	Pb^2+^ Cu^2+^ Cd^2+^ Zn^2+^	Langmuir and Freundlich	[203]
Polymer-functionalized magnetic nanosorbent (PMSC)	Pb^2+^	Langmuir	[181]

## Data Availability

The raw data supporting the conclusions of this article will be made available by the authors upon request.
